# Identification of Gene Signatures and Molecular Mechanisms for Diagnosing Parkinson’s Disease and Nonalcoholic Fatty Liver Disease Using Machine Learning

**DOI:** 10.1155/padi/8731032

**Published:** 2026-05-26

**Authors:** Xuan Chen, Yulin Wu, Yonglai Zhang, Yuli Hou

**Affiliations:** ^1^ Department of Neurology, First Hospital of Shanxi Medical University, Taiyuan, Shanxi, China, sxmu.edu.cn; ^2^ Department of Neurology, Taiyuan City Central Hospital, Peking University First Hospital Taiyuan Branch, The Ninth Clinical Medical College of Shanxi Medical University, Taiyuan, Shanxi, China; ^3^ Changzhi Medical College, Changzhi, Shanxi, China, czmc.com; ^4^ School of Software, North University of China, Taiyuan, Shanxi, China, nuc.edu.cn

**Keywords:** CASP1, CCNA2, immune regulation, INHBE, machine learning, NAFLD, Parkinson’s disease

## Abstract

**Background:**

Parkinson’s disease (PD) is a prevalent neurodegenerative disease, whereas nonalcoholic fatty liver disease (NAFLD) is a common metabolic liver disorder. Growing evidence suggests that NAFLD may affect the central nervous system through the liver–brain axis, potentially contributing to PD, although the underlying molecular mechanisms remain unclear.

**Methods:**

To identify differentially expressed genes (DEGs), transcriptomic data for NAFLD and PD were sourced from the GEO database. Key common candidate genes were screened using protein–protein interaction (PPI) networks, machine learning approaches (LASSO, neural networks, and random forest), and functional enrichment analyses, including GO, KEGG, GSEA, and GSVA. Immune infiltration, TF‐miRNA regulatory networks, and single‐cell RNA sequencing analyses were applied to investigate gene function, immune regulation, and cellular distribution. Candidate drugs were predicted using bioinformatic approaches and validated through molecular docking.

**Results:**

CASP1, CCNA2, and INHBE were identified as three core common candidate genes that may be associated with NAFLD and PD. Involvement of these genes includes inflammatory responses, regulation of the cell cycle, metabolic pathways, and immune microenvironment remodeling. The analysis of the TF‐miRNA network suggested possible regulation by transcription factors CEBPB, BRD4, FOS, and miRNAs such as hsa‐miR‐29b‐1‐5p and hsa‐miR‐128‐3p. Drug prediction and molecular docking identified ethinyl estradiol, mesalamine, and seliciclib as candidate therapeutics, showing strong binding affinity to the core targets.

**Conclusion:**

This study offers a comprehensive elucidation of the molecular ties between NAFLD and PD. The identified core genes and candidate drugs offer theoretical support for potential candidate biomarkers and therapeutic targets in comorbid NAFLD and PD.

## 1. Introduction

Nonalcoholic fatty liver disease (NAFLD) is a common chronic liver disorder, estimated to affect 25%–30% of the adult population globally. It is characterized by abnormal and excessive accumulation of lipids, mainly triglycerides, in the liver, independent of alcohol consumption [1, 2]. NAFLD ranges from simple steatosis, which is not significantly inflammatory, to nonalcoholic steatohepatitis (NASH), which involves both inflammation and damage to hepatocytes. NASH confers a substantially higher risk of liver fibrosis [3, 4]. With the advancement of fibrosis, patients could develop cirrhosis, liver failure, or hepatocellular carcinoma. NAFLD is also closely connected to systemic complications such as metabolic syndrome [5], Type 2 diabetes [6], and cardiovascular diseases [7]. According to recent findings, NAFLD may not only cause liver damage but also affect the central nervous system through the liver–brain axis, suggesting a possible relationship with neurodegenerative disorders [8, 9]. Studies in recent years indicate that NAFLD’s impact extends beyond liver damage. Evidence shows that NAFLD may engage with the central nervous system via the liver–brain axis, thereby altering cognitive performance and neural equilibrium [10]. The liver–brain axis involves the exchange of information between the liver and brain through hormones, metabolites, and immune signaling [10, 11]. Growing evidence indicates that persistent inflammation, disruptions in lipid metabolism, and specific liver‐derived molecules in NAFLD may impact brain function via this axis, especially influencing cognition and mood regulation [12].

Parkinson’s disease (PD) is a frequently occurring neurodegenerative disorder, distinguished mainly by the gradual decline of dopamine neurons in the substantia nigra and the appearance of Lewy bodies [13]. Clinically, PD presents not only with motor symptoms but also with diverse nonmotor manifestations, including cognitive impairment, mood disorders, sleep disturbances, and autonomic dysfunction, which substantially affect quality of life [14, 15]. Although the exact pathogenesis of PD remains incompletely understood, increasing evidence indicates that metabolic abnormalities, immune‐inflammatory responses, environmental factors, and genetic susceptibility all play important roles in its onset and progression [16, 17]. For example, energy metabolism imbalance, mitochondrial dysfunction [18], and chronic low‐grade inflammation [19] may accelerate dopaminergic neuronal damage, while specific genetic mutations or environmental exposures, such as pesticides, can further increase disease risk [20].

Multiple pathways may allow NAFLD to affect the central nervous system’s function. Dysregulated hepatic lipid metabolism and chronic inflammation promote the systemic release of proinflammatory cytokines and metabolic byproducts, increasing blood–brain barrier permeability, activating brain‐resident immune cells, and ultimately inducing neuroinflammation and dopaminergic neuron damage [21]. Moreover, the disruption of the gut–liver–brain axis is considered significant in the comorbidity of NAFLD and PD [22, 23]. NAFLD‐associated gut microbiota dysbiosis can impact the brain through inflammatory, oxidative stress, and metabolic pathways [24], while metabolic abnormalities in PD patients can further promote neuroinflammation and neuronal injury [25], suggesting a complex bidirectional regulatory network between the two conditions. The evidence points to a phenotypic association between NAFLD and PD, but their molecular mechanisms and immune–metabolic interactions still demand comprehensive investigation.

Although previous studies have revealed epidemiological associations and partial pathological mechanisms linking NAFLD and PD [26], the shared molecular drivers, common biomarkers, and interactions within the immune microenvironment remain poorly understood. To bridge these gaps, we integrated transcriptomic data from the GEO database for NAFLD and PD, utilizing bioinformatic strategies to identify genes and gene modules that are differentially expressed in both diseases. We constructed a protein–protein interaction (PPI) network and applied machine learning algorithms to identify key diagnostic genes, examined their associations with immune cell infiltration, and established gene–miRNA and gene–transcription factor (TR) regulatory networks. Furthermore, we confirmed the expression patterns and cellular localization of these crucial genes using single‐cell RNA sequencing data, and assessed potential therapeutic targets through drug prediction and molecular docking studies. By offering new understanding of the comorbidity mechanisms between NAFLD and PD, this study sets the groundwork for potential dual‐purpose diagnostic and treatment strategies.

## 2. Materials and Methods

### 2.1. The Data Source

**FIGURE 1:**
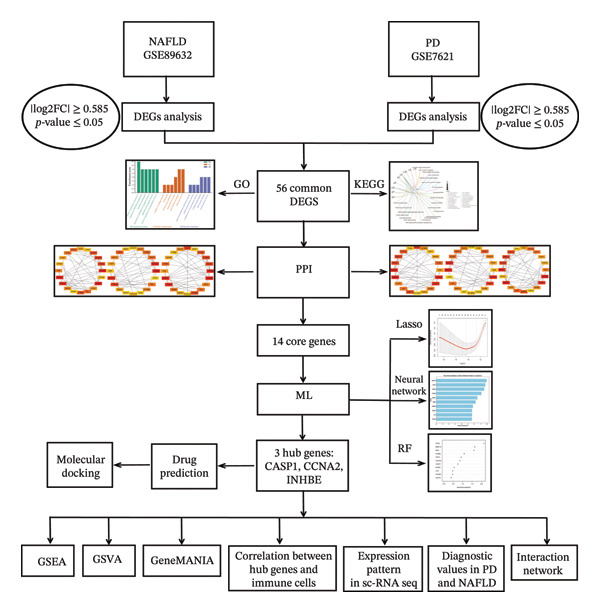
Study flowchart. NAFLD: nonalcoholic fatty liver disease; PD: Parkinson’s disease. DGEs: differentially expressed genes; GO: gene ontology; KEGG: Kyoto Encyclopedia of Genes and Genomes; PPI: protein–protein interaction; ML: machine learning.

For this study, gene expression datasets for NAFLD and PD were obtained from the GEO database (https://www.ncbi.nlm.nih.gov/geo/) to identify differentially expressed genes (DEGs). For NAFLD, the GSE89632 dataset (platform GPL14951) was used, including 24 healthy controls and 19 NAFLD patients. For PD, the GSE7621 dataset (platform GPL570) was used, including 9 healthy controls and 16 PD patients. In addition, GSE164760 was used for external validation of NAFLD, and GSE20141 was used for external validation of PD. The analysis of differential expression was executed using the limma package in R, with thresholds of |log2FC| ≥ 0.585 and an adjusted *p* value < 0.05 to find DEGs associated with the disease. The dataset GSE136103 for NAFLD in single‐cell RNA sequencing analysis had 4 healthy controls and 4 NAFLD patients, while the dataset GSE161045 for PD also had 4 healthy controls and 4 PD patients. Figure 1 illustrates the overall workflow of this study.

### 2.2. Screening and PPI Analysis of Key Targets

For the construction of the PPI network, core targets were first analyzed using the STRING database (https://cn.string-db.org/) and visualized in Cytoscape. The organism was set to human, and the minimum interaction score was set to medium confidence (0.4). Targets with the highest degree in the network were considered key nodes. Subsequently, the CytoHubba plugin was used to further screen core targets based on six algorithms: Closeness, Maximum Clique Centrality (MCC), Degree, Edge Percolated Component (EPC), Radiality, and Stress, in order to identify the most influential proteins within the network.

### 2.3. Machine Learning Analysis

To identify key predictive features, this study employed three machine learning algorithms: Lasso, neural network, and random forest (RF). Lasso was used for feature selection and dimensionality reduction with a random seed set to 123. The neural network used a multilayer perceptron (MLPClassifier) with 50 and 25 nodes in the hidden layers and a maximum of 1000 iterations to train all samples. Feature importance was assessed based on the sum of absolute weights from the input layer to the first hidden layer, and the top 10 genes were selected for further analysis. The RF model was used to further assess predictive performance and feature importance, with a random seed set to 123,456. All models were trained and validated using standard cross‐validation procedures in R, and key features were selected based on their contribution to model performance.

### 2.4. Functional Enrichment Analysis

To explore the potential biological functions and pathways associated with the identified genes, we conducted functional enrichment analyses using multiple approaches. Gene–gene interaction networks were constructed using GeneMANIA. Pathway enrichment analysis was performed via gene set enrichment analysis (GSEA) and gene set variation analysis (GSVA). For both GSEA and GSVA, the gene set file c2.cp.kegg.Hs.symbols.gmt from the Molecular Signatures Database (MSigDB) was employed. GSEA identified pathways significantly enriched in different phenotypic groups, while GSVA was used to assess the variation in pathway activity at the sample level.

### 2.5. Immune Infiltration Analysis

The CIBERSORT algorithm was applied to estimate the relative fractions of immune cell types within the tissue samples. Differences in immune cell composition between disease and control groups were visualized using violin plots. Pearson correlation analysis was performed to assess the relationships between key diagnostic genes and immune cell populations, which were subsequently displayed in a heatmap.

### 2.6. Single‐Cell RNA Sequencing Analysis

This study used single‐cell transcriptome data sourced from the GEO database. The raw data were quality‐controlled to remove low‐quality cells as well as erythrocytes and ribosomal RNA transcripts, followed by log normalization and integration to eliminate batch effects. Dimensionality reduction was performed using Uniform Manifold Approximation and Projection (UMAP), and cell clustering was conducted with the FindClusters function in the Seurat package. Cell‐type annotation was achieved using classical marker genes in combination with the SingleR package.

### 2.7. Molecular Docking

In this study, the three‐dimensional structures of metabolites were obtained from the PubChem database (https://pubchem.ncbi.nlm.nih.gov/), while the structures of core targets were retrieved from the AlphaFold Protein Structure Database (https://alphafold.ebi.ac.uk/). Molecular docking was performed using CB‐Dock2 (http://183.56.231.194:8001/cb-dock2/index.php), which automatically identifies binding sites and docks ligands to targets. The docking results with the lowest binding energies were selected. Visualization and further analysis of the metabolite‐target complexes were conducted using PyMOL and Discovery Studio 2025 Client.

### 2.8. Construction of miRNA–mRNA–TF Interaction Network

We screened candidate miRNAs using the miRWalk database (http://mirwalk.umm.uni-heidelberg.de/) and validated them with the miRDB database (https://mirdb.org/). miRNAs common to both databases were selected for this study. TFs were retrieved from the hTFtarget database (https://guolab.wchscu.cn/hTFtarget/%23;!/). After importing all filtered mRNAs, miRNAs, and TFs, the miRNA‐mRNA‐TF regulatory network was visualized using Cytoscape.

## 3. Results

### 3.1. Identification of Common DEGs in Patients With NAFLD and PD

Differential expression analysis of the NAFLD transcriptome identified a total of 2154 DEGs, including 987 upregulated and 1167 downregulated genes. In the PD transcriptome, 1167 DEGs were detected, comprising 556 upregulated and 611 downregulated genes. Heatmaps were generated to display the expression patterns of the top 30 statistically significant genes in each dataset, providing a clear overview of expression differences across samples (Figures 2(a) and 2(c)). Volcano plots further illustrated the magnitude and statistical significance of gene expression changes (Figures 2(b) and 2(d)). Venn diagram analysis revealed 33 shared upregulated genes and 23 shared downregulated genes between the two datasets (Figures 2(e) and 2(f)), suggesting that these common DEGs may play key roles in the disease‐related mechanisms of NAFLD and PD.

FIGURE 2Identification of differentially expressed genes (DEGs) in NAFLD (GSE89632) and PD (GSE7621) patients. (a) Heatmap of the top 30 upregulated and top 30 downregulated DEGs in GSE89632; (b) volcano plot of all DEGs in GSE89632; (c) heatmap of the top 30 upregulated and top 30 downregulated DEGs in GSE7621; (d) volcano plot of all DEGs in GSE7621; (e) Venn diagram identifies coupregulated DEGs; and (f) Venn diagram identifies codownregulated DEGs.

**Figure:**
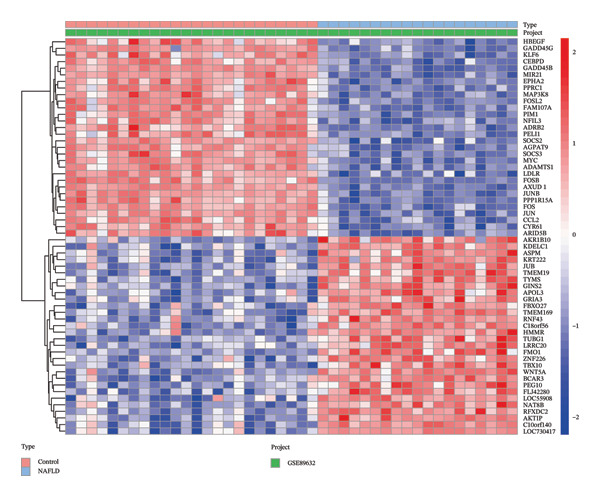
(a)

**Figure:**
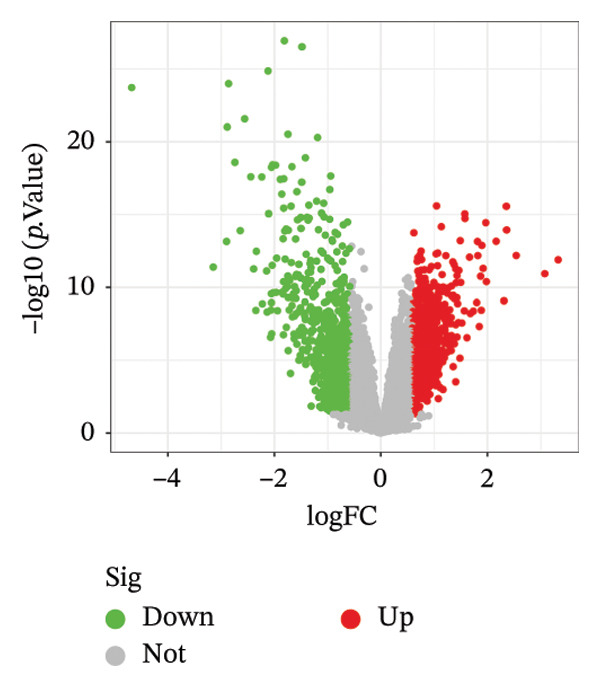
(b)

**Figure:**
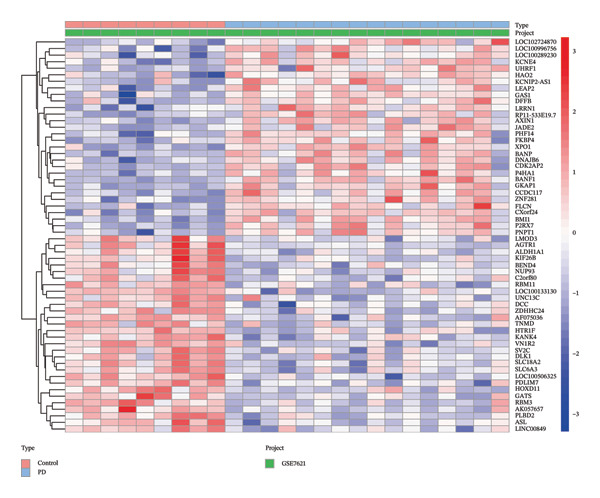
(c)

**Figure:**
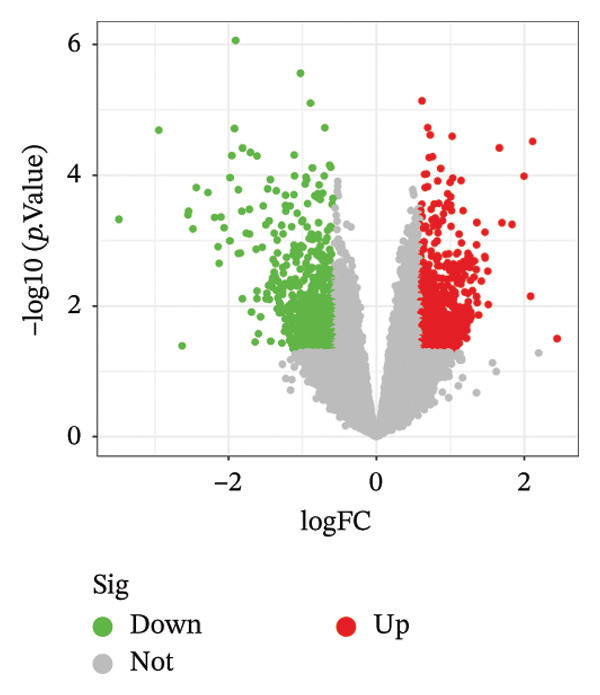
(d)

**Figure:**
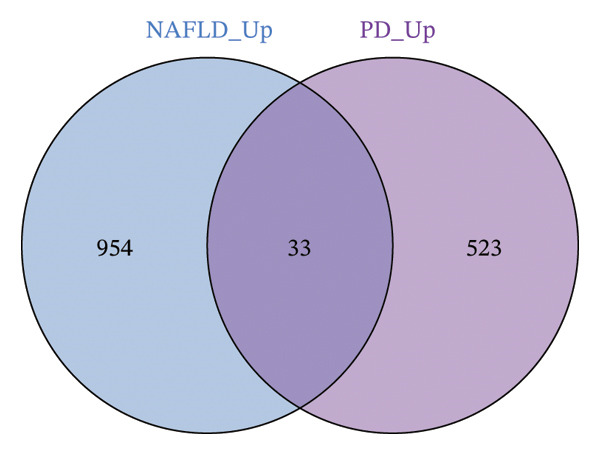
(e)

**Figure:**
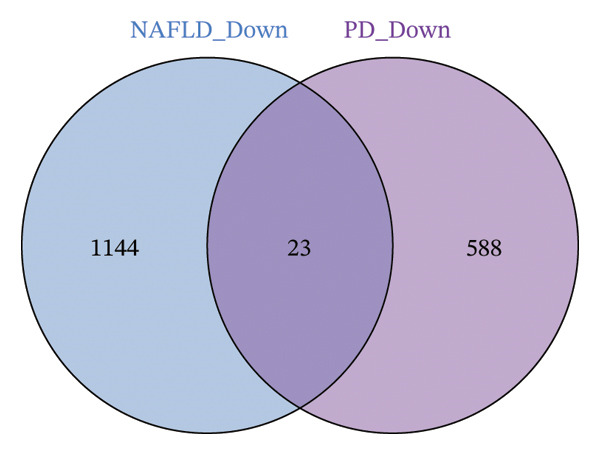
(f)

### 3.2. Gene Ontology (GO) and Kyoto Encyclopedia of Genes and Genomes (KEGG) Enrichment Analysis

GO and KEGG enrichment analyses were performed to explore the potential biological functions and pathways associated with the common DEGs in NAFLD and PD. For the upregulated genes, biological process (BP) terms were primarily related to regulation of fibroblast proliferation, epithelial cell apoptotic process, and positive regulation of proteolysis. Cellular component (CC) terms highlighted collagen trimer complex and desmosome, whereas molecular function (MF) terms were enriched for acyltransferase activity, galactosyltransferase activity, and sugar transmembrane transporter activity (Figure 3(a)). KEGG pathway analysis indicated that these genes were involved in immune and metabolic pathways, including the C‐type lectin receptor, NOD‐like receptor, Ras, and insulin signaling pathway, as well as lipid metabolism‐related pathways such as alpha‐linolenic acid metabolism and glycerolipid metabolism (Figure 3(b)).

FIGURE 3Functional enrichment analysis of common DEGs in NAFLD and PD. (a) GO analysis of upregulated DEGs, showing the top 6 terms with the highest enrichment in BP, CC, and MF; (b) KEGG analysis of upregulated DEGs; (c) GO analysis of downregulated DEGs, showing the top 6 terms with the highest enrichment in BP, CC, and MF; and (d) KEGG analysis of downregulated DEGs.

**Figure:**
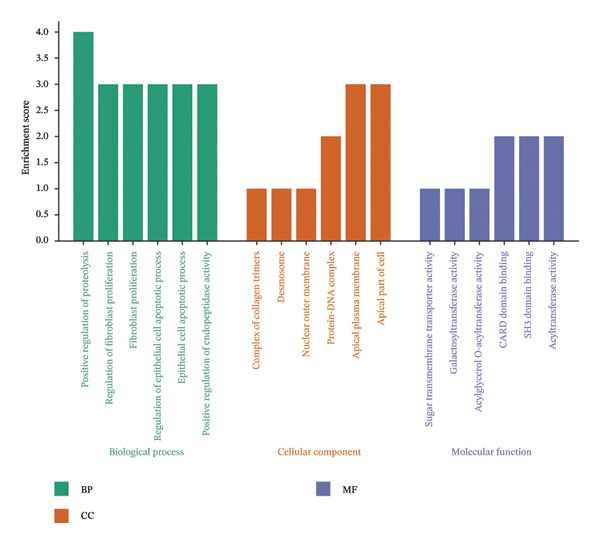
(a)

**Figure:**
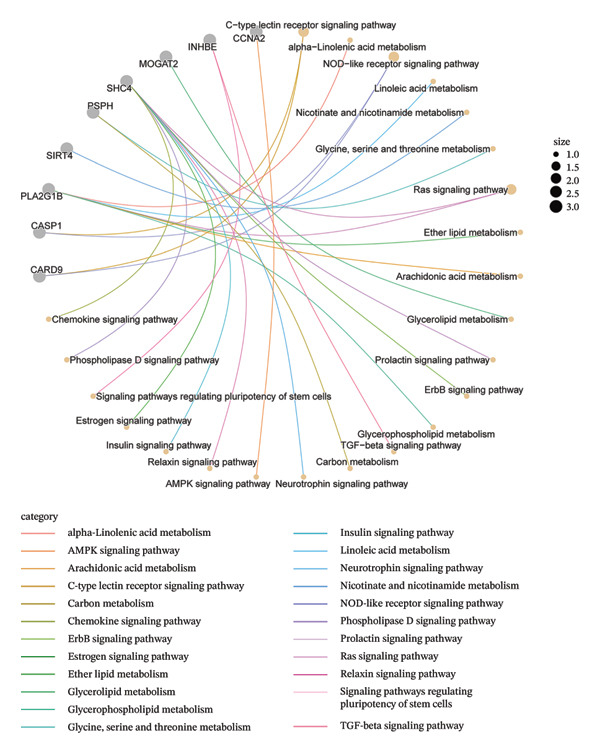
(b)

**Figure:**
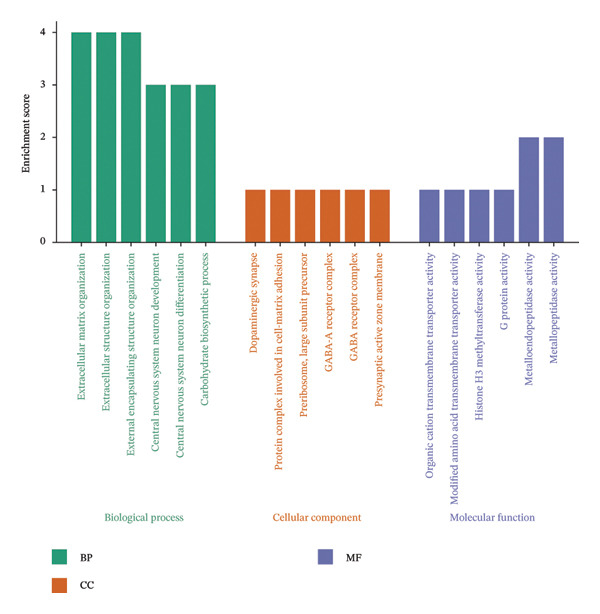
(c)

**Figure:**
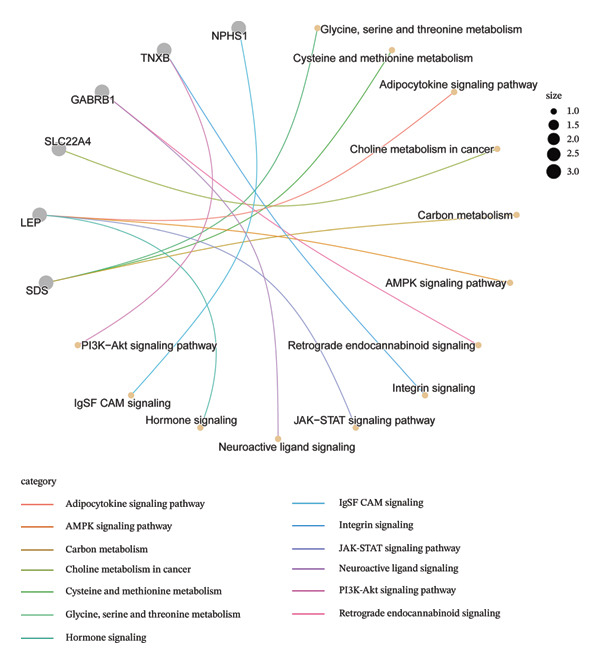
(d)

For the downregulated genes, BP terms were mainly associated with extracellular matrix organization, central nervous system neuron differentiation, and carbohydrate biosynthetic processes. CC terms included dopaminergic synapse, GABA receptor complex, and protein complexes involved in cell‐matrix adhesion, while MF terms were enriched for metallopeptidase activity, histone H3 methyltransferase activity, and organic cation transmembrane transporter activity (Figure 3(c)). KEGG analysis revealed involvement in metabolic and signaling pathways, including glycine, serine, and threonine metabolism, adipocytokine, AMPK, PI3K‐Akt signaling pathway, and retrograde endocannabinoid signaling (Figure 3(d)). These results suggest that the shared DEGs in NAFLD and PD may contribute to immune regulation, lipid and amino acid metabolism, neuronal development, and synaptic function, highlighting potential molecular links between metabolic liver disease and neurodegenerative processes.

### 3.3. PPI Network Analysis

In this study, a PPI network of 56 DEGs was constructed and visualized using the STRING database (Figure 4(a)) to intuitively display the interactions among genes. To further prioritize candidate therapeutic targets, we applied six centrality algorithms based on network topology: Closeness, MCC, Degree, EPC, Radiality, and Stress. These algorithms were used to quantify the relative importance of each node in the interaction network (Figures 4(b), 4(c), 4(d), 4(e), 4(f), and 4(g)). By intersecting the top 20 high‐scoring genes from each method, 14 core targets were ultimately identified. These genes exhibit high connectivity and play key regulatory roles in the network (Figure 4(h)), providing priority candidate targets for subsequent studies.

FIGURE 4PPI network construction. (a) Visualization of the PPI network for the DEGs using STRING database; hub genes determined through multiple CytoHubba algorithms, including (b) Closeness, (c) MCC, (d) Degree, (e) EPC, (f) Radiality, and (g) Stress, to identify the most influential genes in the network; (h) Venn diagram depicting the common hub genes identified across the different scoring methods, representing a consensus set of core genes for further functional exploration.

**Figure:**
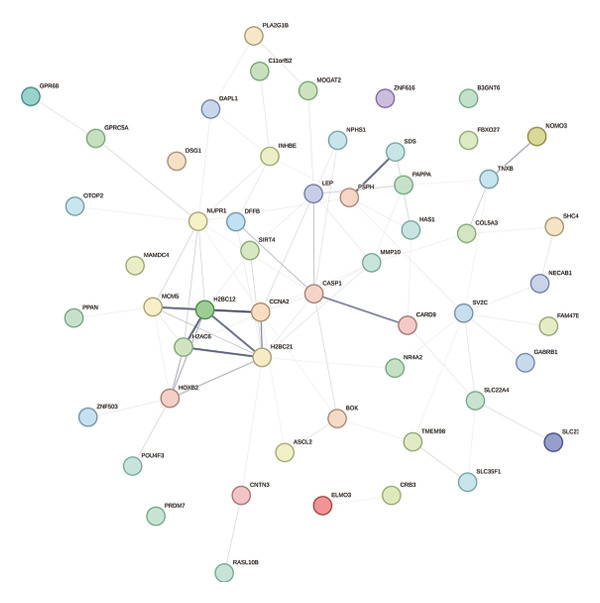
(a)

**Figure:**
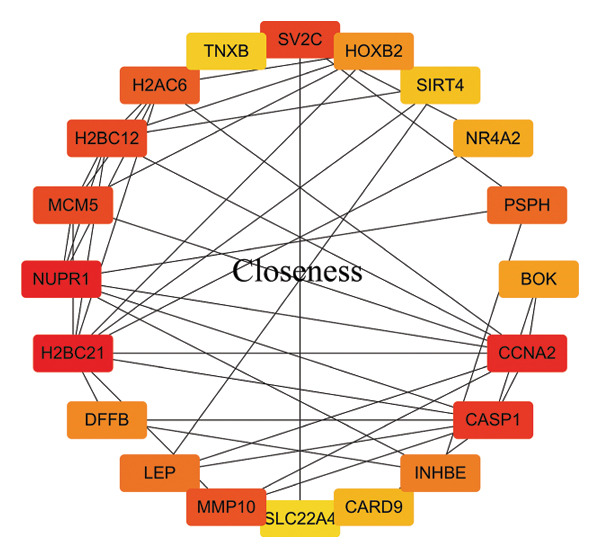
(b)

**Figure:**
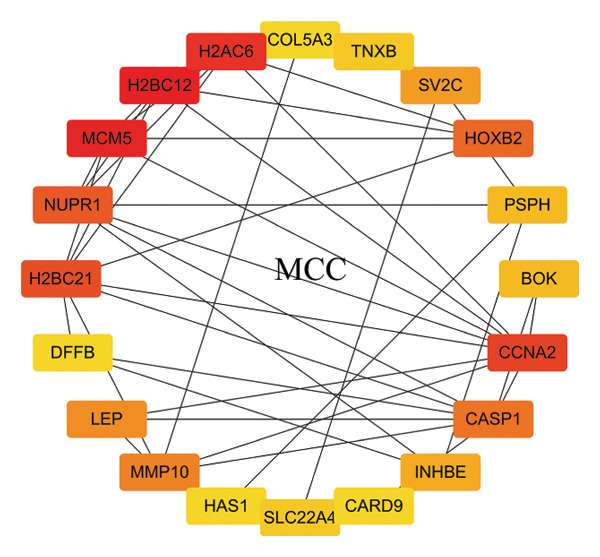
(c)

**Figure:**
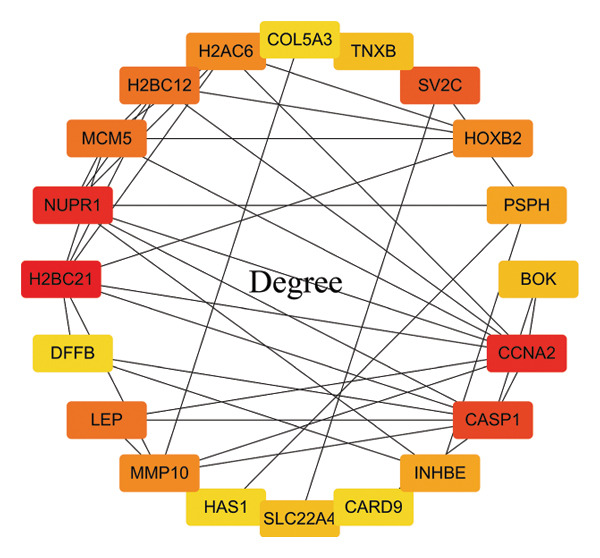
(d)

**Figure:**
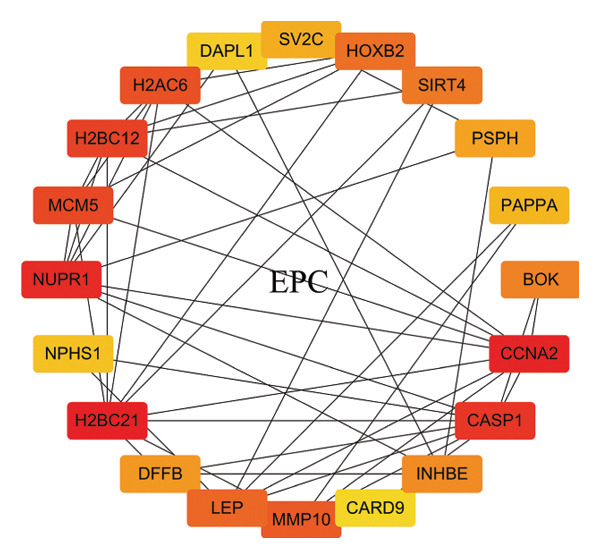
(e)

**Figure:**
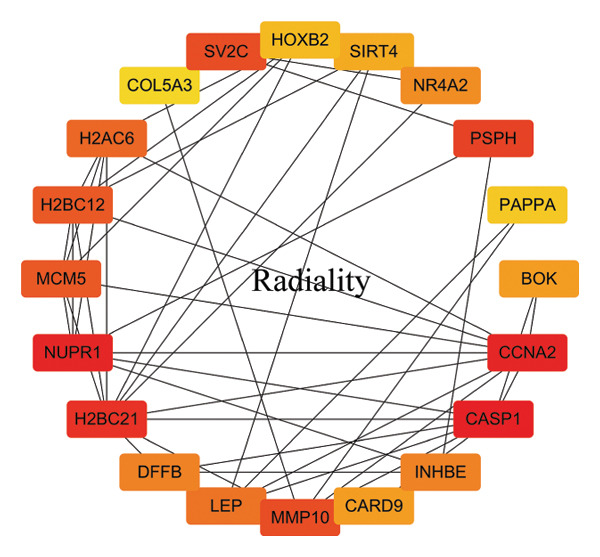
(f)

**Figure:**
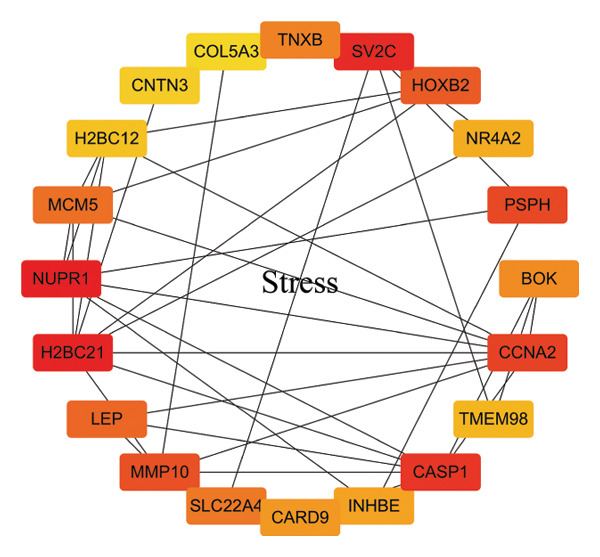
(g)

**Figure:**
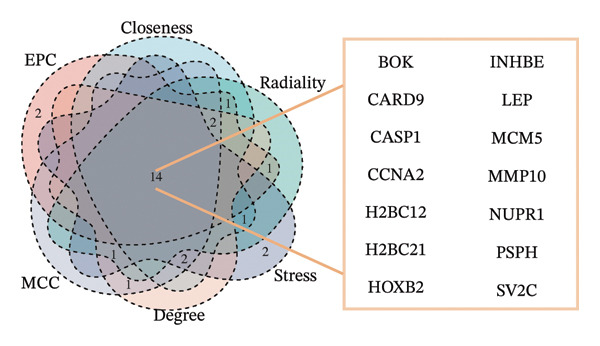
(h)

### 3.4. Core Gene Selection and Validation via Machine Learning

To further identify key genes, DEGs were analyzed using Lasso, neural network, and RF methods. In the NAFLD dataset, five core targets were selected using Lasso regression (Figure 5(a)). The neural network and RF analyses identified the top 10 genes based on feature importance scores (Figures 5(b) and 5(c)). By intersecting these results and visualizing them with a Venn diagram, four core targets were ultimately identified: caspase‐1 (CASP1), cyclin A2 (CCNA2), inhibin subunit beta E (INHBE), and NUPR1 (Figure 5(g)). Similarly, in the PD dataset, five core targets were selected using Lasso regression (Figure 5(d)). The neural network and RF analyses identified the top 10 genes based on feature importance scores (Figures 5(e) and 5(f)). By intersecting these results and visualizing them with a Venn diagram, five core targets were ultimately identified: CASP1, CCNA2, INHBE, MMP10, and SV2C (Figure 5(h)). Subsequently, the genes common to both model results (CASP1, CCNA2, and INHBE) were selected as candidate markers for further analysis (Figure 5(i)).

FIGURE 5Feature gene selection using machine learning algorithms. (a) Feature gene selection in GSE89632 using Lasso; (b) top 10 important genes identified in GSE89632 using a neural network algorithm; (c) feature gene selection in GSE89632 using random forest (RF); (d) feature gene selection in GSE7621 using Lasso; (e) top 10 important genes identified in GSE7621 using a neural network algorithm; (f) feature gene selection in GSE7621 using RF; (g) Venn diagram of core genes identified by three machine learning methods in NAFLD; (h) Venn diagram of core genes identified by three machine learning methods in PD; and (i) intersection of NAFLD and PD target genes after machine learning selection.

**Figure:**
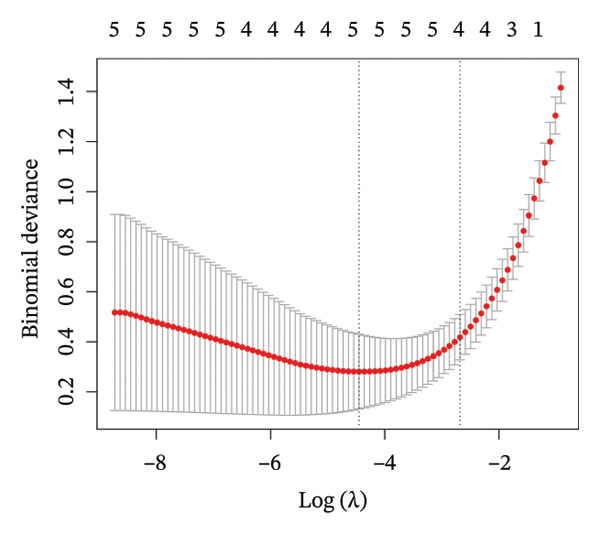
(a)

**Figure:**
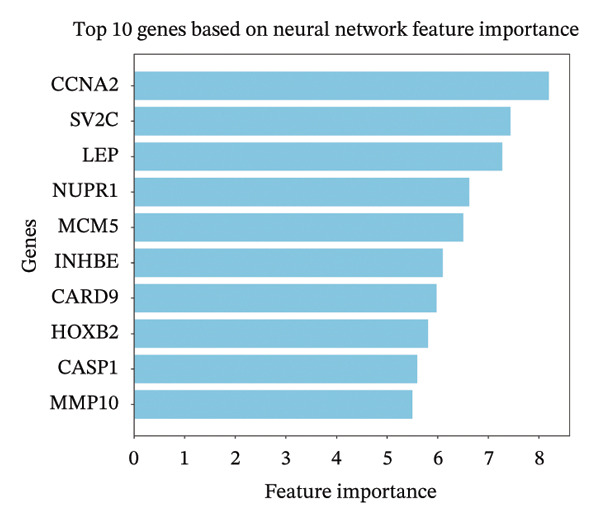
(b)

**Figure:**
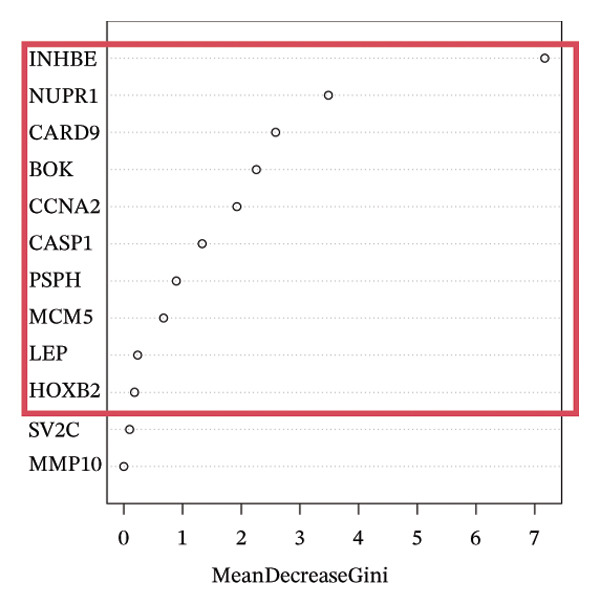
(c)

**Figure:**
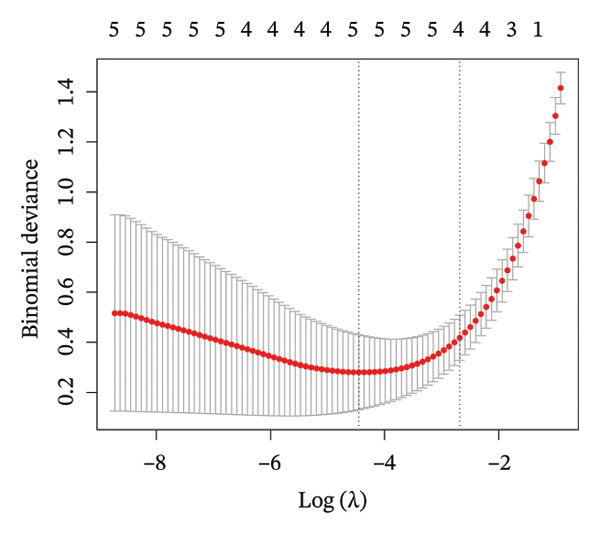
(d)

**Figure:**
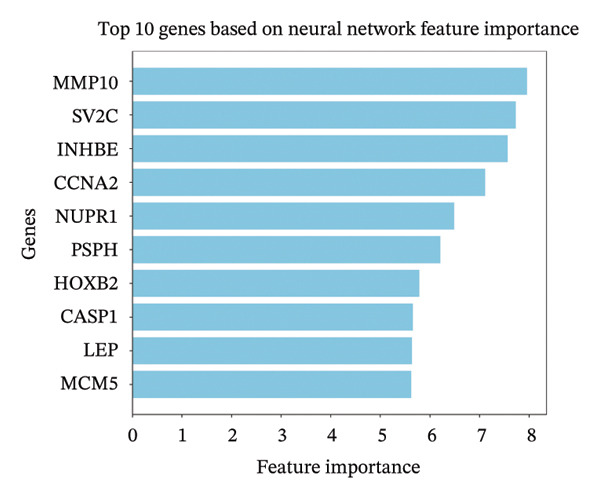
(e)

**Figure:**
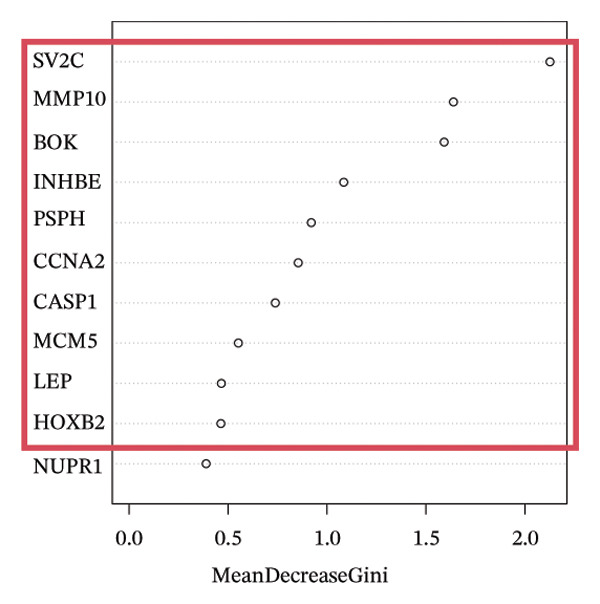
(f)

**Figure:**
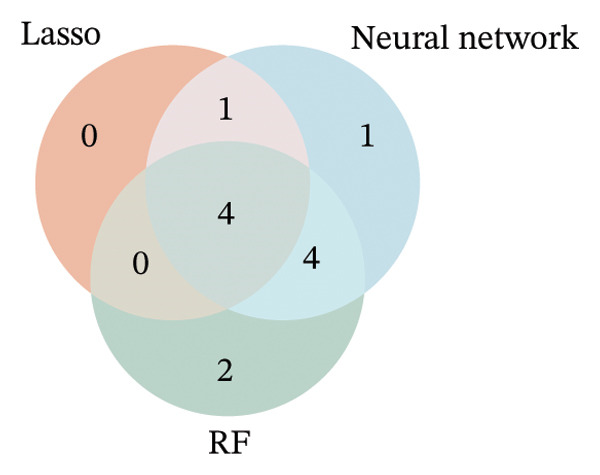
(g)

**Figure:**
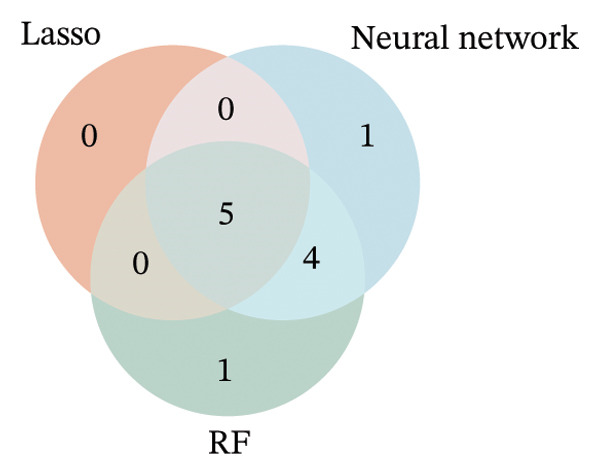
(h)

**Figure:**
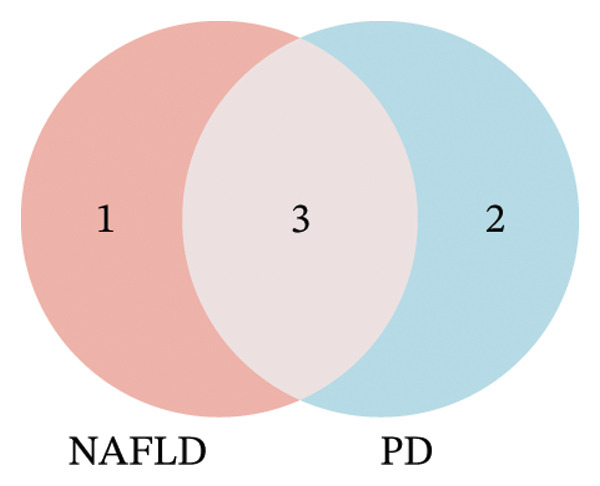
(i)

### 3.5. Expression and Diagnostic Value of Core Genes

We further evaluated the expression levels of the three core genes in the NAFLD and PD datasets. As shown in Figures 6(a) and 6(b), all three core genes were significantly upregulated in the disease groups (*p* < 0.05). Subsequently, receiver operating characteristic (ROC) curve analysis was performed across different datasets to assess the diagnostic performance of these genes and the constructed model. The results demonstrated that, in both the NAFLD and PD datasets, the three genes achieved area under the curve (AUC) values greater than 0.8 (Figures 6(c) and 6(d)), indicating good diagnostic efficacy for both diseases. In addition, we validated the core candidate genes using external datasets, and the results showed significant differential expression (Figure S1).

FIGURE 6ROC curves were used to evaluate the diagnostic efficacy of key genes and the model. The expression levels of CASP1, CCNA2, and INHBE in (a) the NAFLD dataset and (b) the PD dataset. (c) ROC curves of the three genes in the NAFLD dataset; (d) ROC curves of the three genes in the PD dataset.

**Figure:**
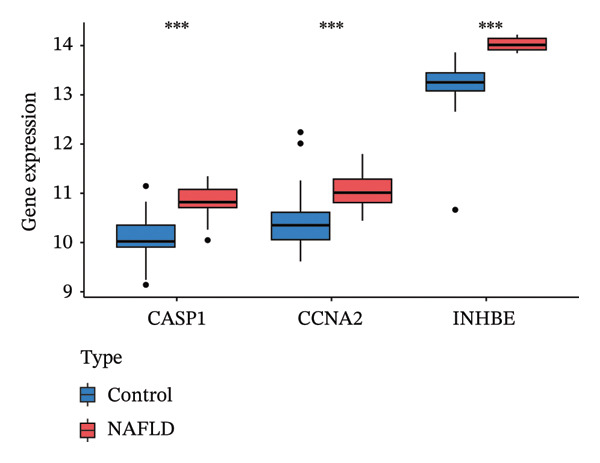
(a)

**Figure:**
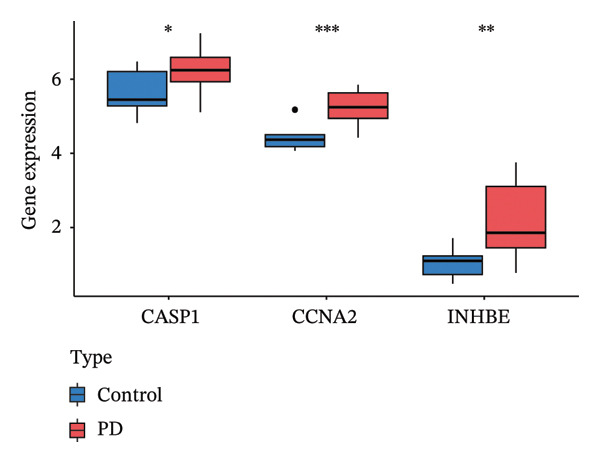
(b)

**Figure:**
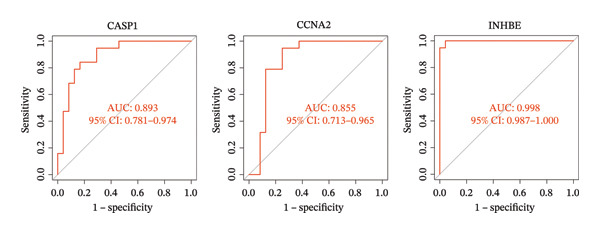
(c)

**Figure:**
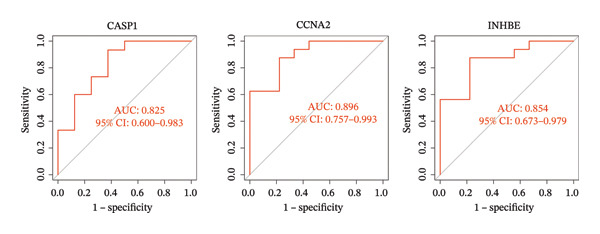
(d)

### 3.6. Functional Analysis of Core Genes in NAFLD

GeneMANIA analysis showed that CCNA2 is primarily involved in cell cycle regulation, including the G1 DNA damage checkpoint and the G2/M transition; INHBE is associated with transmembrane receptor serine/threonine kinase signaling and SMAD protein phosphorylation, suggesting a potential role in fibrotic signaling; and CASP1 is enriched in inflammatory responses, such as IL‐1β production and inflammasome formation, indicating its involvement in inflammation‐mediated liver injury. Overall, these three genes may contribute to the pathogenesis of NAFLD through cell cycle regulation, fibrotic signaling, and inflammatory pathways (Figure 7(a)).

FIGURE 7Functional analysis of the three genes in the NAFLD dataset. (a) GeneMANIA network analysis of the three genes; (b) GSEA, with samples grouped by high and low gene expression; and (c) GSVA functional enrichment analysis.

**Figure:**
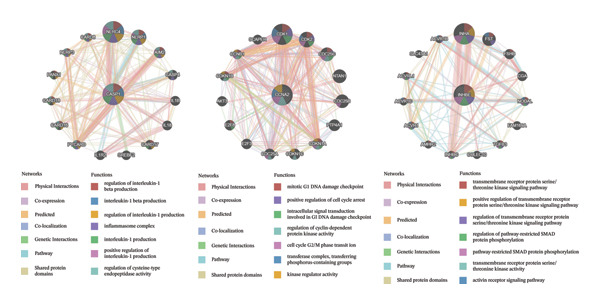
(a)

**Figure:**
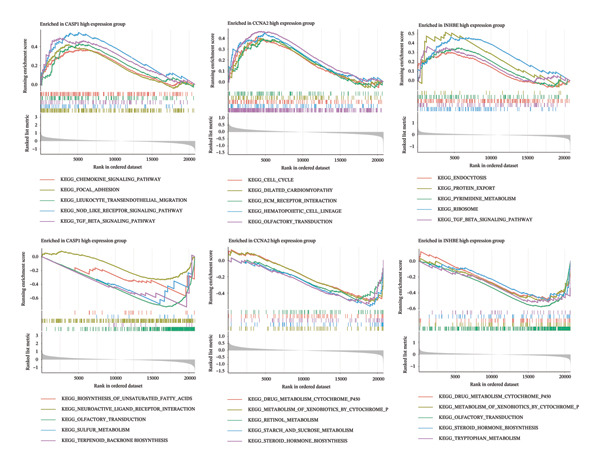
(b)

**Figure:**
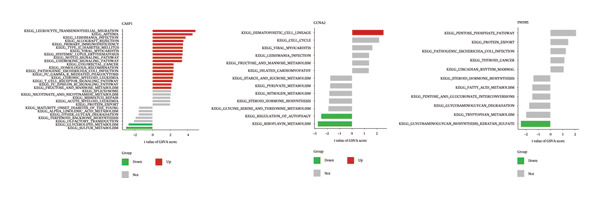
(c)

GSEA and GSVA showed that the three core genes are involved in multiple key biological pathways in NAFLD. High expression of CASP1 is mainly enriched in chemokine signaling and NOD‐like receptor signaling pathways, suggesting a central role in inflammatory responses and immune cell migration. High expression of INHBE is primarily associated with endocytosis and TGF‐β signaling pathways, indicating its potential involvement in fibrotic signaling and intercellular communication in the liver. High expression of CCNA2 is mainly involved in the cell cycle and extracellular matrix–receptor interactions, suggesting an important role in hepatocyte proliferation, regeneration, and liver tissue remodeling (Figures 7(b) and 7(c)).

### 3.7. Functional Analysis of Core Genes in PD

In the PD dataset, GSEA and GSVA showed that the three core genes are involved in distinct biological pathways. CASP1 high expression was enriched in immune‐related pathways, such as cytokine–receptor interaction, Toll‐like receptor signaling, and natural killer cell‐mediated cytotoxicity, indicating its key role in inflammation and immune responses; low expression was associated with neural signaling pathways, including calcium signaling and long‐term potentiation. INHBE high expression was mainly linked to neural signaling and metabolic pathways, such as calcium signaling and neuroactive ligand–receptor interaction, while low expression was associated with metabolic and cell proliferation pathways, including adipocytokine signaling and chronic myeloid leukemia–related pathways. CCNA2 high expression was enriched in cell cycle and tumor‐related pathways, including cell cycle, pancreatic cancer, and chronic myeloid leukemia, whereas low expression was mainly associated with metabolic and cardiovascular‐related pathways. Therefore, these three genes may contribute to PD pathogenesis through inflammation and immune regulation, neural signaling, and cell cycle/proliferation control, providing insights into disease mechanisms and potential therapeutic targets (Figures 8(a) and 8(b)).

FIGURE 8Functional analysis of the three genes in the PD dataset. (a) GSEA, with samples grouped by high and low gene expression, and (b) GSVA functional enrichment analysis.

**Figure:**
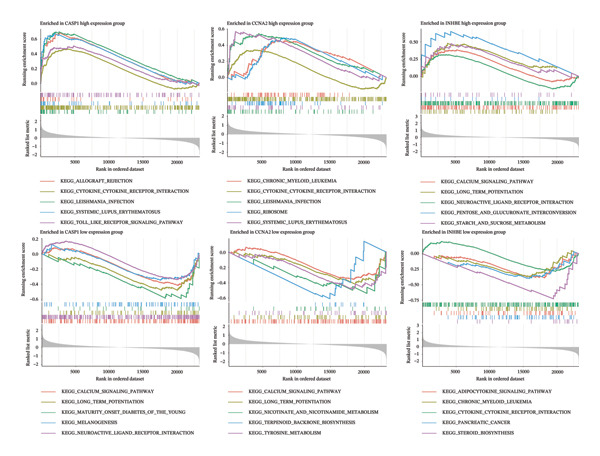
(a)

**Figure:**
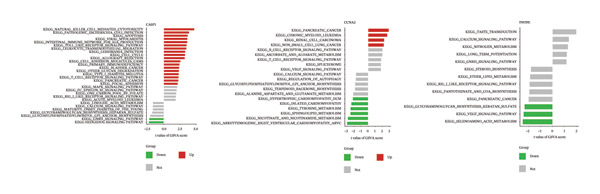
(b)

### 3.8. Immune Cell Infiltration

As shown in Figures 9(a) and 9(e), the immune cell profiles differed significantly between the disease and control groups. Figure 9(b) and 9(f) show heatmaps of correlations among different immune cells, further highlighting the significant differences in immune profiles between the disease and control groups, as well as the interactions among various immune cell types. Compared with the control group, NAFLD tissues exhibited increased levels of CD4 memory resting T cells, γδ T cells, M1 macrophages, M2 macrophages, resting dendritic cells, and resting mast cells, whereas naive B cells, plasma cells, activated NK cells, monocytes, activated dendritic cells, and neutrophils were decreased (Figure 9(c)). In the PD group, CD8 T cells and γδ T cells were decreased compared with the normal group, whereas resting NK cells were increased (Figure 9(g)). Notably, the immune cell profiles in both NAFLD and PD tissues exhibited significant alterations, suggesting that both diseases are associated with remodeling of the immune microenvironment. These changes may be related to chronic inflammation, tissue injury, and metabolic abnormalities: under disease conditions, some adaptive immune cells may become exhausted or migrate to sites of inflammation, while certain innate immune cells may increase compensatorily, thereby influencing disease progression and tissue repair.

FIGURE 9Analysis of immune cell infiltration. (a, e) Stacked bar charts showing the proportions of immune cell infiltration in tissues of NAFLD and PD patients; (b, f) heatmaps of correlations between different immune cells in tissues of NAFLD and PD patients; (c, g) box plots comparing immune cell proportions between NAFLD and PD patients and the control group (^∗^ *p* < 0.05, ^∗∗^ *p* < 0.01, and ^∗∗∗^ *p* < 0.001); and (d, h) correlation analysis between infiltrating immune cells and key immune‐related genes in tissues of NAFLD and PD patients.

**Figure:**
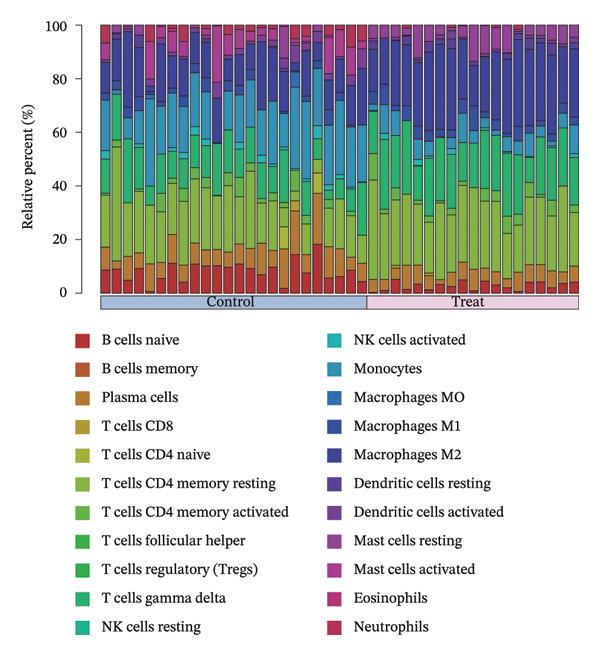
(a)

**Figure:**
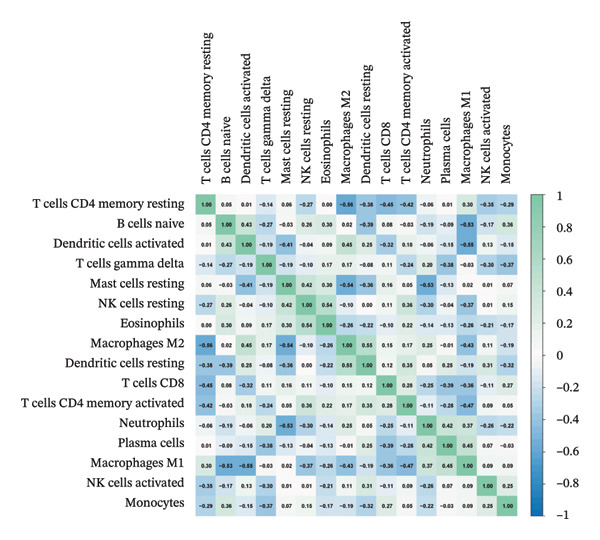
(b)

**Figure:**
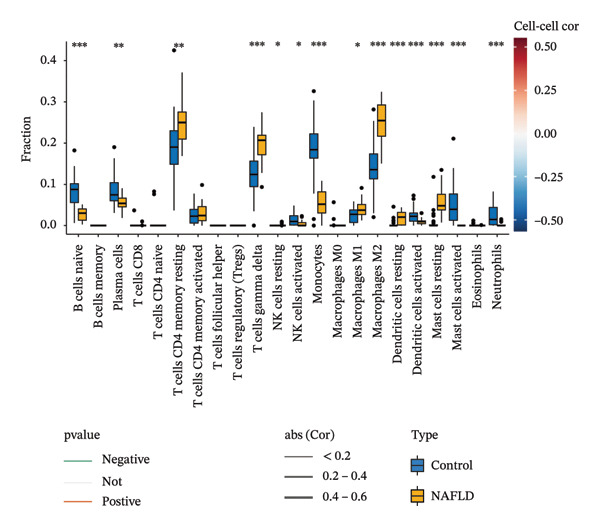
(c)

**Figure:**
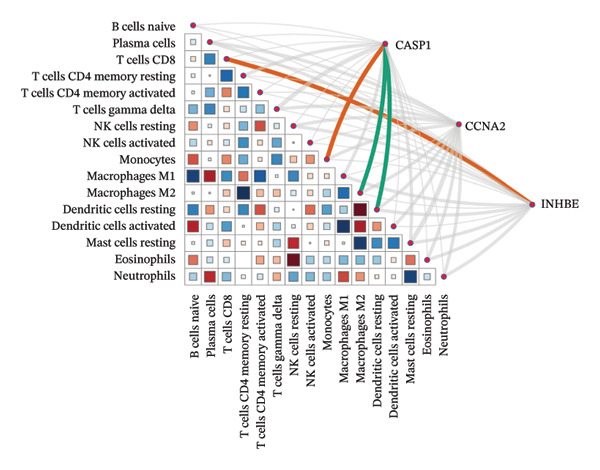
(d)

**Figure:**
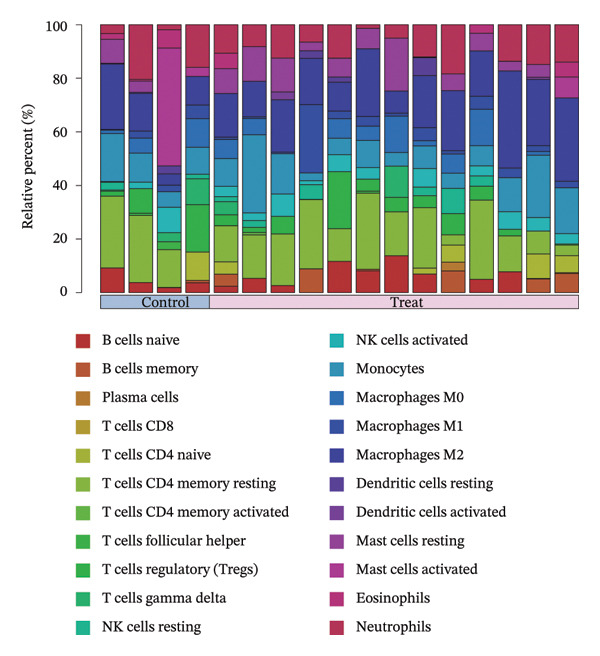
(e)

**Figure:**
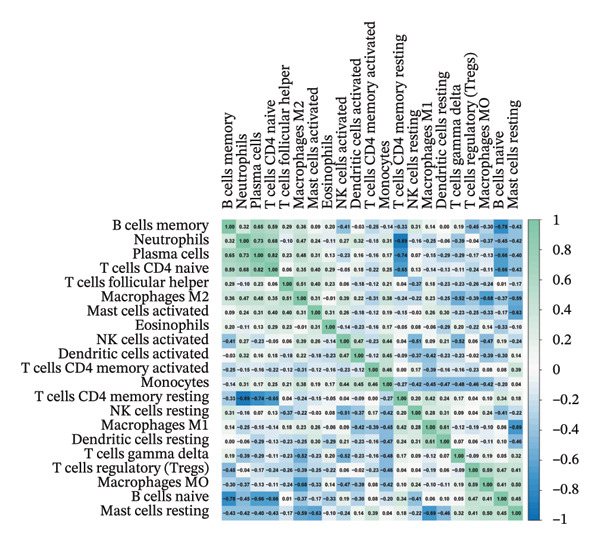
(f)

**Figure:**
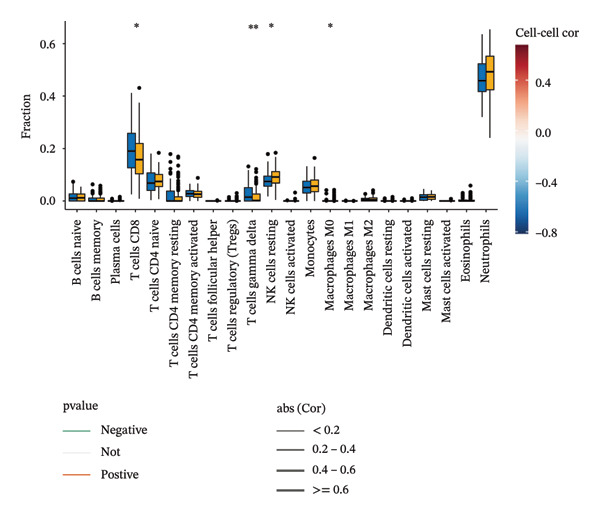
(g)

**Figure:**
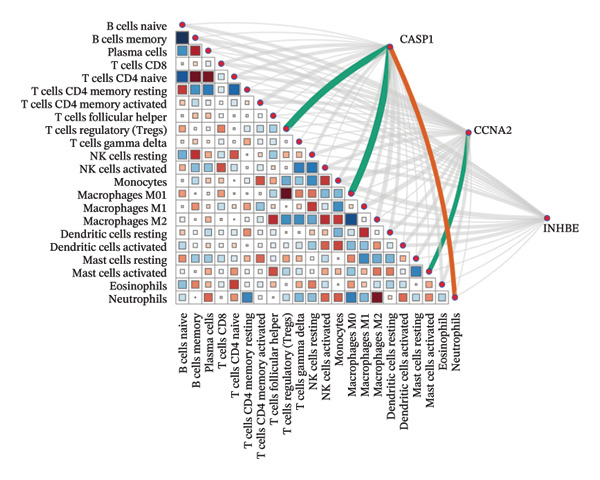
(h)

In addition, we analyzed the relationship between core immune–related genes and the composition of immune cells in NAFLD and PD patients. In NAFLD, CASP1 was positively correlated with monocytes and negatively correlated with M2 macrophages and resting dendritic cells, while INHBE was associated with CD8 T cells (Figure 9(d)). In PD, CASP1 was negatively correlated with regulatory T cells (Tregs) and M0 macrophages but positively correlated with neutrophils and CCNA2 was negatively correlated with activated mast cells (Figure 9(h)). These findings suggest that core immune genes may modulate the immune microenvironment in NAFLD and PD through distinct pathways.

### 3.9. Single‐Cell Sequencing Data–Based Verification of the Expression of Hub Diagnostic Genes

After quality control of the single‐cell data in NAFLD (Figure S2), eight distinct cell populations were identified through cell annotation: T cells, NK cells, hepatocytes, monocytes, endothelial cells, B cells, smooth muscle cells, and macrophages (Figure 10(a)). UMAP plots illustrated the expression and distribution of the three core genes across these cell populations. CASP1 was mainly expressed in monocytes, T cells, and NK cells (Figure 10(c)). CCNA2 showed low expression in T cells and INHBE was barely expressed (Figures 10(e) and 10(g)).

FIGURE 10Single‐cell analysis. (a, b) UMAP plots of different cell populations after cell annotation in NAFLD and PD patients; (c–h) UMAP plots showing the expression and distribution of key diagnostic genes across different cell populations.

**Figure:**
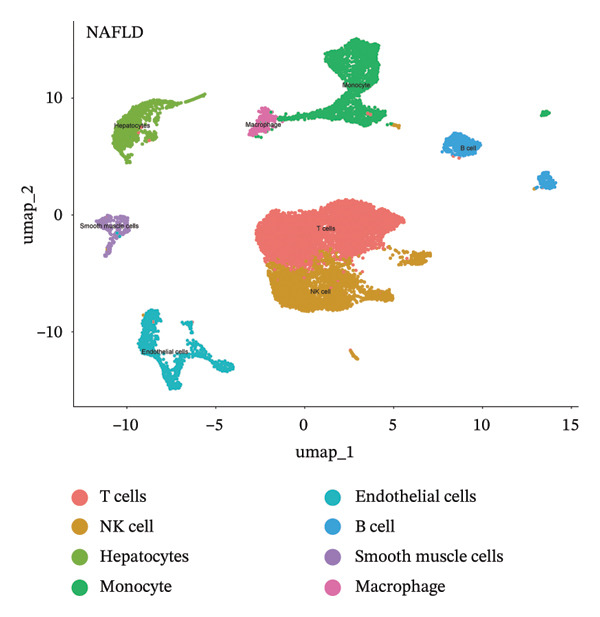
(a)

**Figure:**
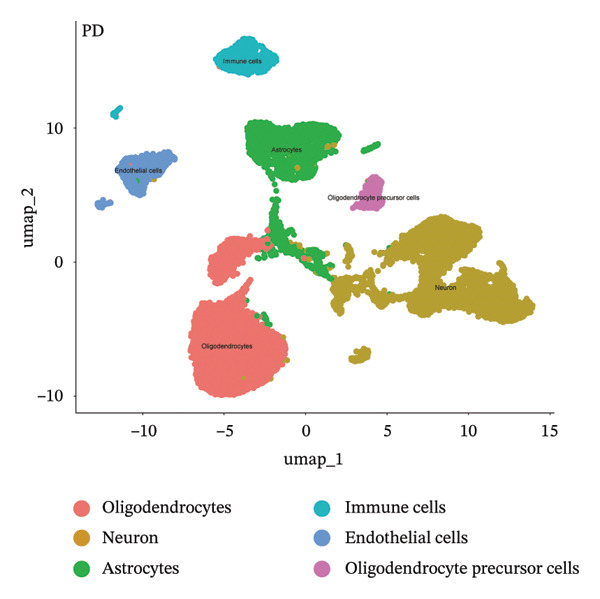
(b)

**Figure:**
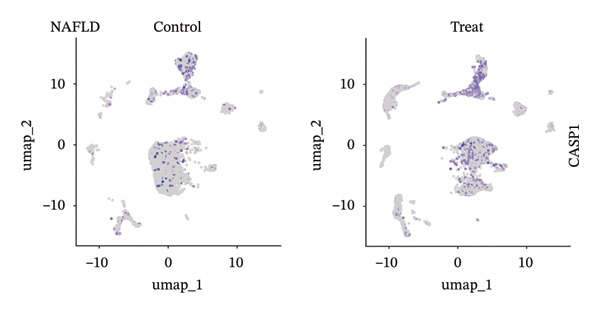
(c)

**Figure:**
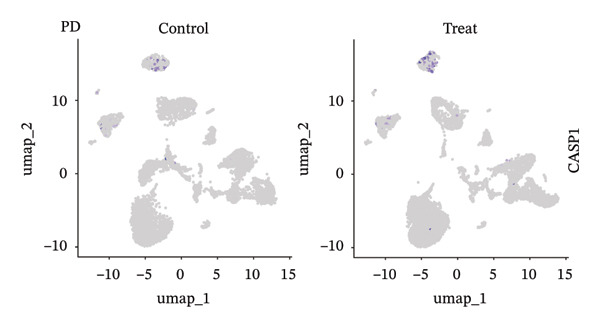
(d)

**Figure:**
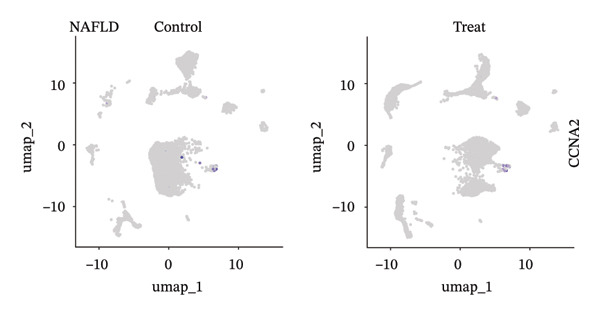
(e)

**Figure:**
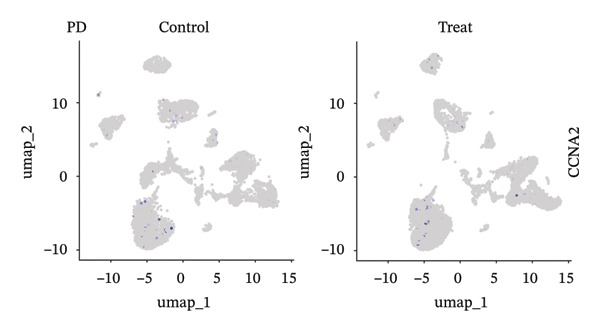
(f)

**Figure:**
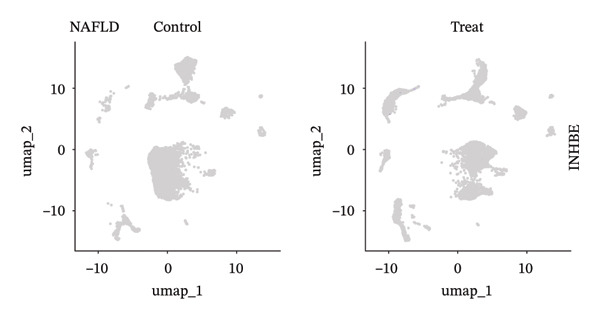
(g)

**Figure:**
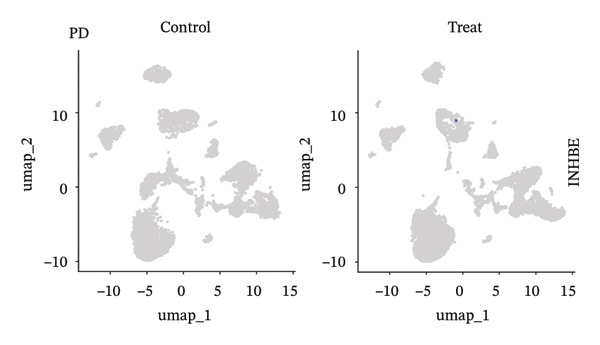
(h)

After quality control of the single‐cell data in PD (Figure S3), six distinct cell populations were identified: oligodendrocytes, neurons, astrocytes, immune cells, endothelial cells, and oligodendrocyte precursor cells (Figure 10(b)). UMAP plots showed the expression and distribution of the three core genes across these cell populations. CASP1 was mainly expressed in immune cells (Figure 10(d)). CCNA2 showed low expression in oligodendrocytes and INHBE was almost not expressed (Figures 10(f) and 10(h)). The near absence of INHBE expression in the major cell populations of both NAFLD and PD suggests that this gene has limited activity in these mature cells and may primarily function in rare cell types or under specific pathological conditions.

### 3.10. Regulatory Network Analysis of Core Genes With miRNAs and TFs

In the miRWalk and miRDB databases, CASP1, INHBE, and CCNA2 were predicted to be regulated by 20, 23, 112, and 27 miRNAs, respectively (Figure 11(a)). In the hTFtarget database, TFs with predicted scores greater than 2 were selected and CASP1, INHBE, and CCNA2 were predicted to be regulated by 12, 41, and 36 TFs, respectively. Among them, CEBPB, POLR2A, SPI1, BRD4, and FOS were predicted to coregulate all three core genes (Figure 11(b)), suggesting that these TFs may play key roles in their coordinated regulation.

FIGURE 11Construction of miRNA–mRNA–TF regulatory networks. (a) Hub genes–miRNAs regulatory network and (b) hub genes–TFs regulatory network. Hub genes are shown as red circles, transcription factors as light blue diamonds, and miRNAs as yellow shapes.

**Figure:**
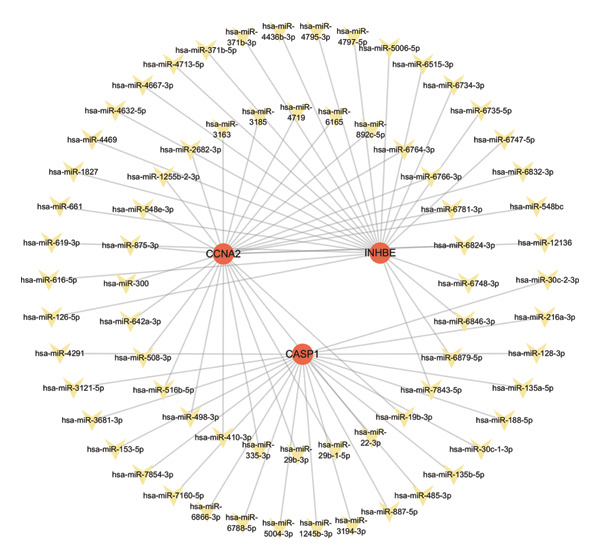
(a)

**Figure:**
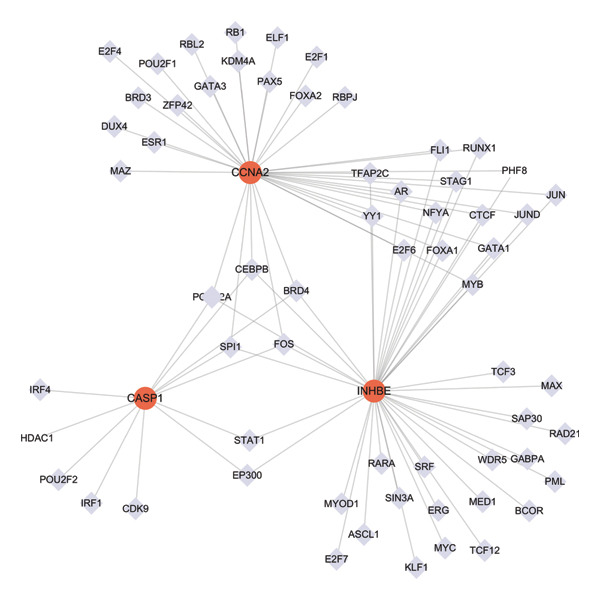
(b)

### 3.11. Drug Prediction and Molecular Docking

To explore potential therapeutic strategies, we predicted candidate drugs using Enrich (Table S1) and identified the top three candidates: ethinyl estradiol, mesalamine, and seliciclib. Molecular docking results showed that the binding energies of these three drugs with the three core targets were all below −5 kcal/mol (Figure 12(a)), indicating strong binding affinity to the target proteins. Figures 12(b), 12(c), 12(d), 12(e), 12(f), 12(g), 12(h), 12(i), and 12(j) illustrate the binding pockets and key interaction sites of each drug with their respective targets, visually depicting the binding modes and critical interacting residues. Therefore, these results suggest that these drugs have potential target‐specific activity and could serve as possible interventions for the treatment of NAFLD and PD.

FIGURE 12Molecular docking results. (a) Heatmap of docking binding energies; (b–d) docking conformation of CASP1 with seliciclib, ethinyl estradiol, and mesalamine; (e–g) docking conformation of CCNA2 with seliciclib, ethinyl estradiol, and mesalamine; and (h–j) docking conformation of INHBE with seliciclib, ethinyl estradiol, and mesalamine.

**Figure:**
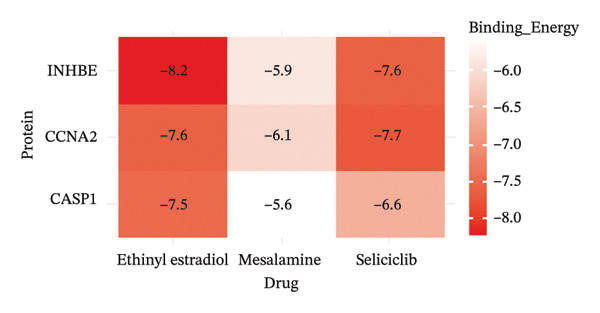
(a)

**Figure:**
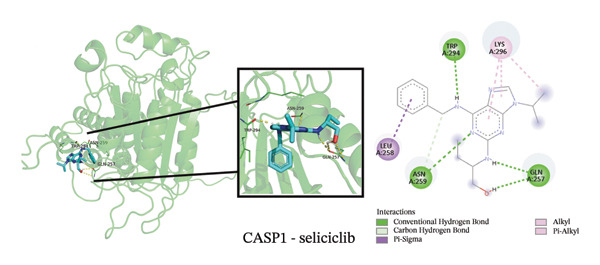
(b)

**Figure:**
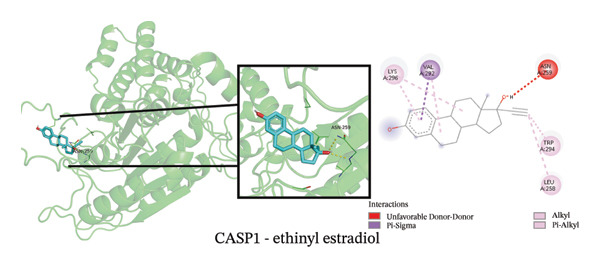
(c)

**Figure:**
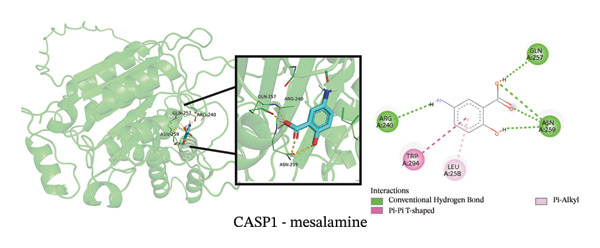
(d)

**Figure:**
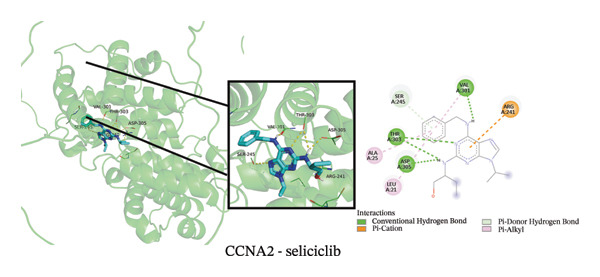
(e)

**Figure:**
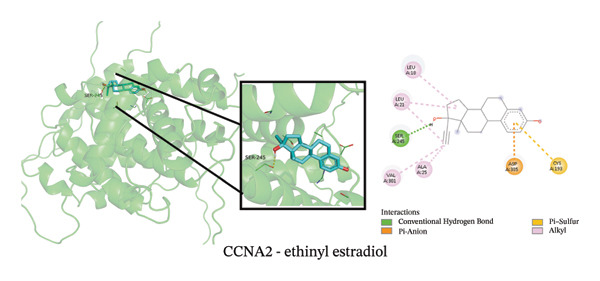
(f)

**Figure:**
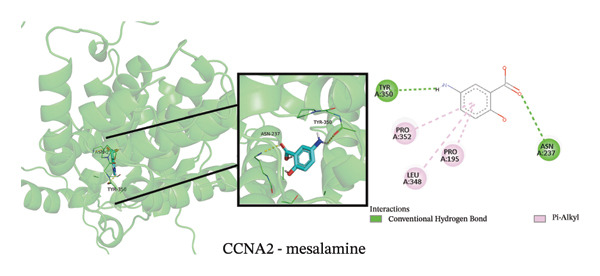
(g)

**Figure:**
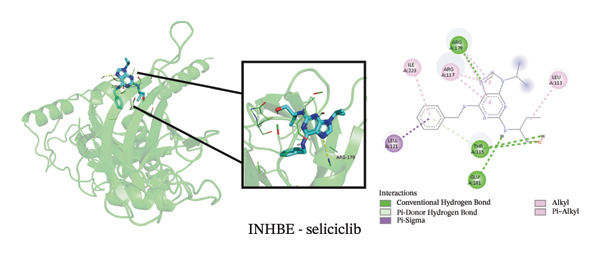
(h)

**Figure:**
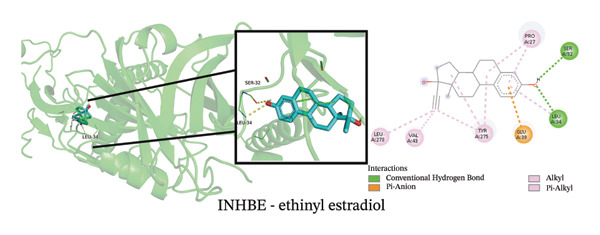
(i)

**Figure:**
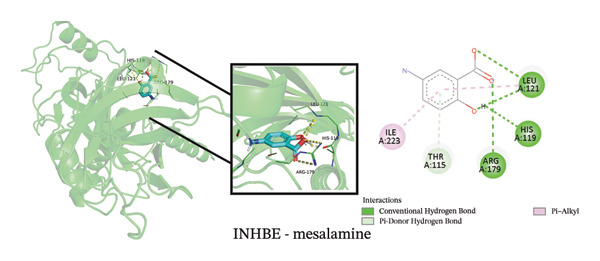
(j)

## 4. Discussion

Increasing epidemiological and experimental evidence indicates that NAFLD is not only a liver‐specific metabolic disorder but can also affect central nervous system function through metabolic dysfunction, chronic inflammation, and immune dysregulation [21, 27], thereby increasing the risk of neurodegenerative diseases [28]. PD, the second most common neurodegenerative disorder, is closely associated with systemic inflammation, immune imbalance, and metabolic abnormalities [29, 30]. The recently proposed “liver–brain axis” provides a new framework to explore the potential link between NAFLD and PD although the underlying molecular mechanisms remain unclear.

By integrating differential expression analysis, coexpression network analysis, and machine learning, this study identified CASP1, CCNA2, and INHBE as important common candidate genes for PD and NAFLD. The roles of these genes in inflammation, cell cycle, and metabolic pathways suggest they may be essential molecular links between NAFLD and PD. In the inflammasome pathway, CASP1 is an essential effector that aids in the maturation and release of IL‐1β and IL‐18, playing a significant role in chronic inflammation and immune responses [31]. Prior studies have revealed that the progression of NAFLD is closely linked to the sustained activation of the NLRP3–CASP1 inflammasome, which heightens hepatocyte lipotoxicity and stimulates liver inflammation [32]. In the context of PD, the activation of CASP1 in an abnormal manner has been reported to cause microglia‐related neuroinflammation and damage to dopaminergic neurons [33, 34]. Notably, systemic inflammatory factors can affect the central nervous system via the blood–brain barrier or the vagus nerve, suggesting that CASP1‐mediated inflammation in NAFLD may accelerate neurodegeneration in PD through the peripheral‐central immune axis.

CCNA2 is a key regulator of the cell cycle, primarily controlling the G1/S and G2/M transitions. Mature neurons are typically in a permanent cell cycle arrest, and aberrant re‐entry into the cell cycle is considered a major mechanism underlying neuronal apoptosis and neurodegeneration [35]. In PD, abnormal activation of cell cycle‐related molecules in dopaminergic neurons has been linked to neuronal loss [36]. In NAFLD, dysregulated CCNA2 expression is associated with imbalanced hepatocyte proliferation, inflammation‐related liver injury, and disease progression [37]. In this study, CCNA2 was found to be aberrantly expressed in both PD and NAFLD, suggesting that cell cycle dysregulation may serve as a key bridge linking metabolic inflammation to neurodegeneration.

INHBE, a member of the TGF‐β superfamily, is mainly expressed in the liver and functions as a hepatokine involved in energy metabolism, lipid homeostasis, and insulin sensitivity [38]. Recent studies have shown that INHBE is markedly upregulated in NAFLD and metabolic syndrome and is closely associated with liver inflammation and lipid dysregulation. Notably, liver‐derived factors can act on the brain via humoral pathways, influencing neuroinflammation and neuronal function [39]. In this study, INHBE was also found to be associated with PD, suggesting that liver metabolic dysregulation may contribute to PD development through INHBE‐mediated endocrine signaling, providing molecular evidence for the “liver–brain axis.”

Although CCNA2 and INHBE show significant differential expression in NAFLD and PD in bulk RNA‐seq, their expression is less prominent than CASP1 at the single‐cell level. This may be because CCNA2 is mainly highly expressed in actively dividing cells, while most mature somatic cells are in a cell‐cycle‐arrested state, resulting in generally low expression in conventional single‐cell tissue maps and noticeable expression only in actively proliferating cell subpopulations [40]. INHBE is a liver‐specific secreted factor primarily expressed in hepatocytes and secreted into the circulation to regulate energy metabolism and lipid homeostasis, so its detection frequency in standard single‐cell RNA‐seq cell types is low [38, 41]. This notion is further supported by emerging evidence demonstrating that activin E, encoded by INHBE, functions as a hepatokine that modulates adipose tissue lipolysis and systemic metabolic homeostasis [42]. In addition, low‐abundance transcripts are prone to dropout due to capture or amplification biases [43]. Therefore, CCNA2 and INHBE are less prominently expressed than CASP1 in single‐cell maps.

This study preliminarily explored the immune link between NAFLD and PD, revealing significant remodeling of the immune microenvironment in both diseases. In NAFLD, the balance between adaptive and innate immune cells is disrupted, suggesting that chronic inflammation and metabolic abnormalities may drive immune cell activation, exhaustion, or migration to inflammatory sites. Activation of monocytes and macrophages can release proinflammatory factors, inducing local inflammation and promoting dysregulated hepatic lipid metabolism [44]. Meanwhile, changes in T cell populations may further modulate the functions of B cells and other immune cells, forming a complex immune regulatory network that influences tissue repair and disease progression [45]. Further analysis indicated that core immune‐related genes may play crucial roles in the comorbidity of NAFLD and PD by regulating inflammatory signaling, immune cell activation, and intercellular communication. These findings suggest that inflammation and immune imbalance are key mechanisms underlying the interaction between NAFLD and PD, providing a molecular basis for their comorbidity.

MiRNAs primarily regulate post‐transcriptional gene expression, while TFs control target gene transcription, both playing crucial roles in various BPs and diseases. Therefore, we constructed a miRNA‐mRNA‐TF competitive regulatory network targeting three core immune‐related genes. Within this network, we identified hsa‐miR‐29b‐1‐5p, hsa‐miR‐335‐3p, hsa‐miR‐128‐3p, and the TFs CEBPB, POLR2A, SPI1, BRD4, and FOS. MiR‐335‐5p has been reported to be significantly downregulated in the serum of PD patients as well as in animal and cellular models [46, 47], suggesting its involvement in inflammation and neurodegenerative pathology. Hsa‐miR‐128‐3p is also downregulated in platelets of PD patients, serving as a potential PD‐related molecular signal [48]. The TF CEBPB shows weakly positive expression changes in inflammation‐related genes under PD conditions [49], indicating a role in neuroinflammation regulation. BRD4 has been shown to play a key role in liver diseases such as fibrosis and is regulated by miR‐29a, contributing to inflammatory and fibrotic processes [50]. Additionally, FOS, as an immediate early gene, is closely associated with neuronal stress, inflammation, and apoptosis. Its upregulation in PD transcriptome analyses may reflect activation of inflammatory or neuronal responses [51].

Although treatments for NAFLD and PD have been continuously developed, their efficacy remains limited and side effects persist, highlighting the need for novel intervention strategies. In this study, enrich analysis identified ethinyl estradiol, mesalamine, and seliciclib as potential candidate drugs, and molecular docking confirmed their high binding affinity to core targets. Ethinyl estradiol, a synthetic estrogen, may improve lipid metabolism dysregulation and chronic inflammation in NAFLD by modulating metabolic and inflammatory pathways [52]. Mesalamine, an anti‐inflammatory agent widely used to control intestinal inflammation, possesses immunomodulatory and anti‐inflammatory properties, suggesting potential in regulating neuroinflammation in PD. Seliciclib, a CDK inhibitor, can regulate the cell cycle and inflammatory responses, potentially affecting hepatocyte proliferation and neuronal stress responses [53]. The target‐binding characteristics of these three drugs provide not only a theoretical basis for pharmacological interventions in NAFLD and PD but also potential directions for the development of future small‐molecule therapies. However, the clinical applicability of these candidate drugs should be interpreted with caution. Although molecular docking indicated favorable binding affinities, these compounds have not been approved for the treatment of NAFLD or PD, and their potential for drug repurposing remains uncertain. Ethinyl estradiol can modulate the blood–brain barrier [54], whereas seliciclib exhibits a low brain‐to‐blood concentration ratio [55], indicating limited brain penetration. In addition, potential systemic side effects, such as headache, nausea, and vomiting [56, 57], should be considered. Therefore, the findings of this study should be regarded as preliminary and require further in vivo and in vitro experimental validation to confirm their therapeutic potential.

This study has several limitations. First, it primarily relies on multiomics data analysis and bioinformatic predictions, with limited clinical validation; thus, the conclusions require further confirmation in prospective cohorts or clinical samples. Second, although we identified potential molecular links between NAFLD and PD, the precise causal mechanisms and the roles of key genes within the neuroimmuneendocrine axis remain to be experimentally validated.

## 5. Conclusion

This study systematically identified three key common candidate genes (CASP1, CCNA2, and INHBE), revealing the potential molecular connections between NAFLD and PD. Functional analyses showed that these genes are not only involved in inflammatory responses, cell cycle regulation, and metabolic dysregulation but also play critical roles in remodeling the immune microenvironment, suggesting that inflammation and immune imbalance may be important mechanisms underlying the interaction between the two diseases. In addition, TF‐miRNA regulatory network analysis revealed the potential roles of multiple TFs (such as CEBPB, BRD4, and FOS) and miRNAs (such as hsa‐miR‐29b‐1‐5p and hsa‐miR‐128‐3p) in regulating the expression of these core genes, providing new perspectives for molecular intervention. Drug prediction and molecular docking analyses further identified candidate drugs, including ethinyl estradiol, mesalamine, and seliciclib, which exhibited strong binding affinity to the core targets, providing a theoretical basis for future small‐molecule interventions and combination therapy strategies.

## Author Contributions

The study’s design, data collection, analysis, interpretation, and the drafting of the first manuscript were all conducted by Xuan Chen, Yonglai Zhang, and Yulin Wu. Xuan Chen, Yonglai Zhang, and Yulin Wu handled data interpretation and made revisions to the manuscript to enrich its intellectual content. Xuan Chen, Yonglai Zhang, and Yuli Hou contributed to the study by designing it, searching the literature, analyzing and interpreting the data, and revising the manuscript for intellectual content.

## Funding

This study was supported by the Science, Technology, and Innovation Medical Center of the Taiyuan Bureau of Science and Technology (Grant no. 202209).

## Disclosure

The manuscript’s final version has received approval from all authors.

## Ethics Statement

All procedures in the study were conducted in accordance with the ethical principles of the Declaration of Helsinki. As the analysis involved only publicly available, deidentified data, it was exempt from additional ethical review.

## Consent

The authors have nothing to report.

## Conflicts of Interest

The authors declare no conflicts of interest.

## Supporting Information

Additional supporting information can be found online in the Supporting Information section.

## Supporting information


**Supporting Information** Figure S1. The expression of core candidate genes was validated using independent external datasets. Figure S2. Quality control, dimensionality reduction, clustering, and annotation of NAFLD single‐cell sequencing data. Figure S3. Quality control, dimensionality reduction, clustering, and annotation of PD single‐cell sequencing data. Table S1. Predicted candidate drugs for NAFLD and PD.

## Data Availability

The data that support the findings of this study are available from the corresponding author upon reasonable request.
