# Supplementary material for: Identification of Gene Signatures and Molecular Mechanisms for Diagnosing Parkinson’s Disease and Nonalcoholic Fatty Liver Disease Using Machine Learning
Source: Parkinsons Dis. 2026 May 26;2026:8731032. doi: 10.1155/padi/8731032 (PMC13202728; doi:10.1155/padi/8731032)
Supplement: Supplementary file 1 — Supporting Information Figure S1. The expression of core candidate genes was validated using independent external datasets. Figure S2. Quality control, dimensionality reduction, clustering, and annotation of NAFLD single‐cell sequencing data. Figure S3. Quality control, dimensionality reduction, clustering, and annotation of PD single‐cell sequencing data. Table S1. Predicted candidate drugs for NAFLD and PD. [file PADI-2026-8731032-s001.docx]

**Identification of gene signatures and molecular mechanisms for diagnosing Parkinson's disease and non-alcoholic fatty liver disease using machine learning**

**Fig. S1** The expression of core candidate genes was validated using independent external datasets.

**Fig. S2**. Quality control, dimensionality reduction, clustering, and annotation of NAFLD single-cell sequencing data.

**Fig. S3.** Quality control, dimensionality reduction, clustering, and annotation of PD single-cell sequencing data.

**Table S1.** Predicted candidate drugs for NAFLD and PD


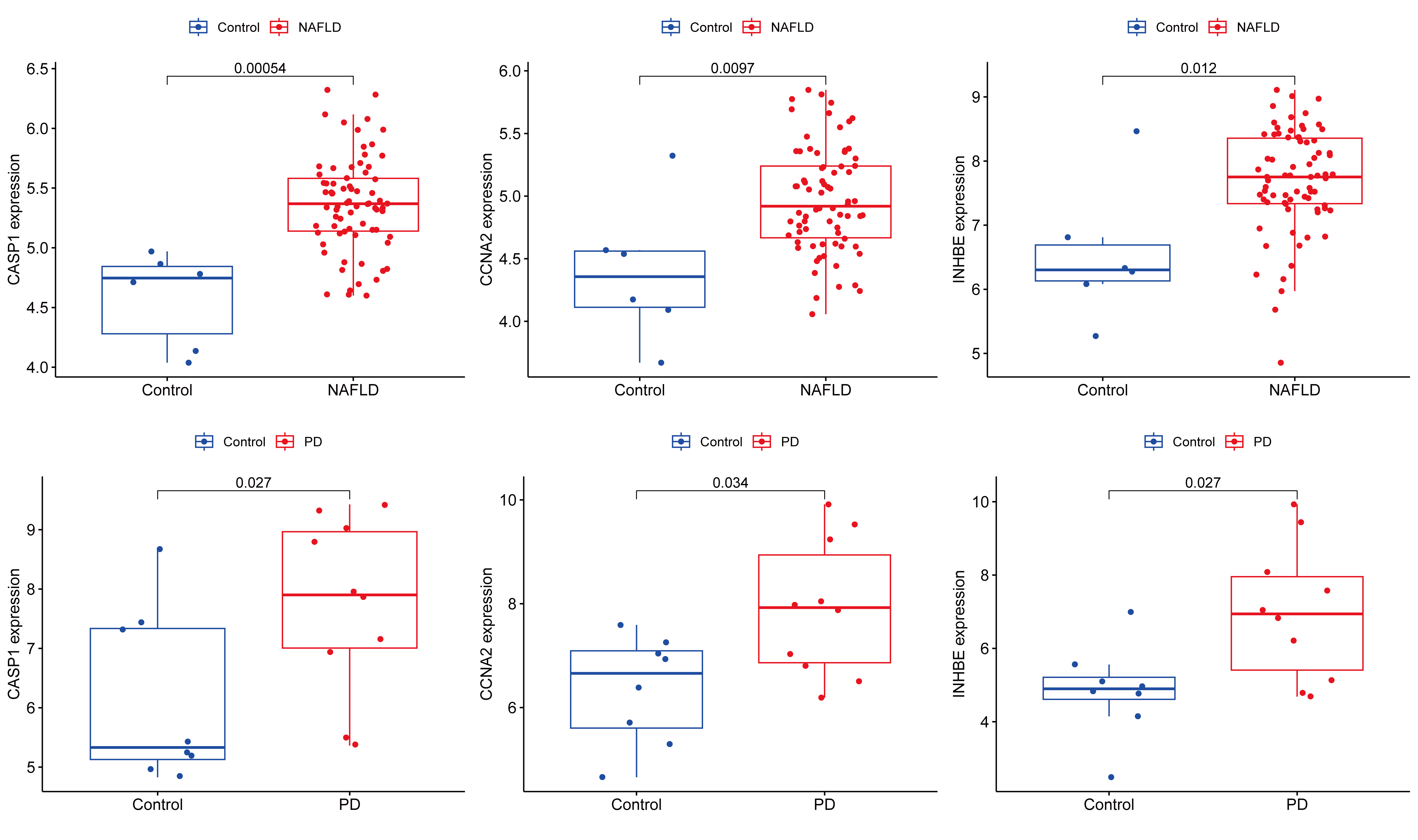


**Fig. S1** The expression of core candidate genes was validated using independent external datasets.


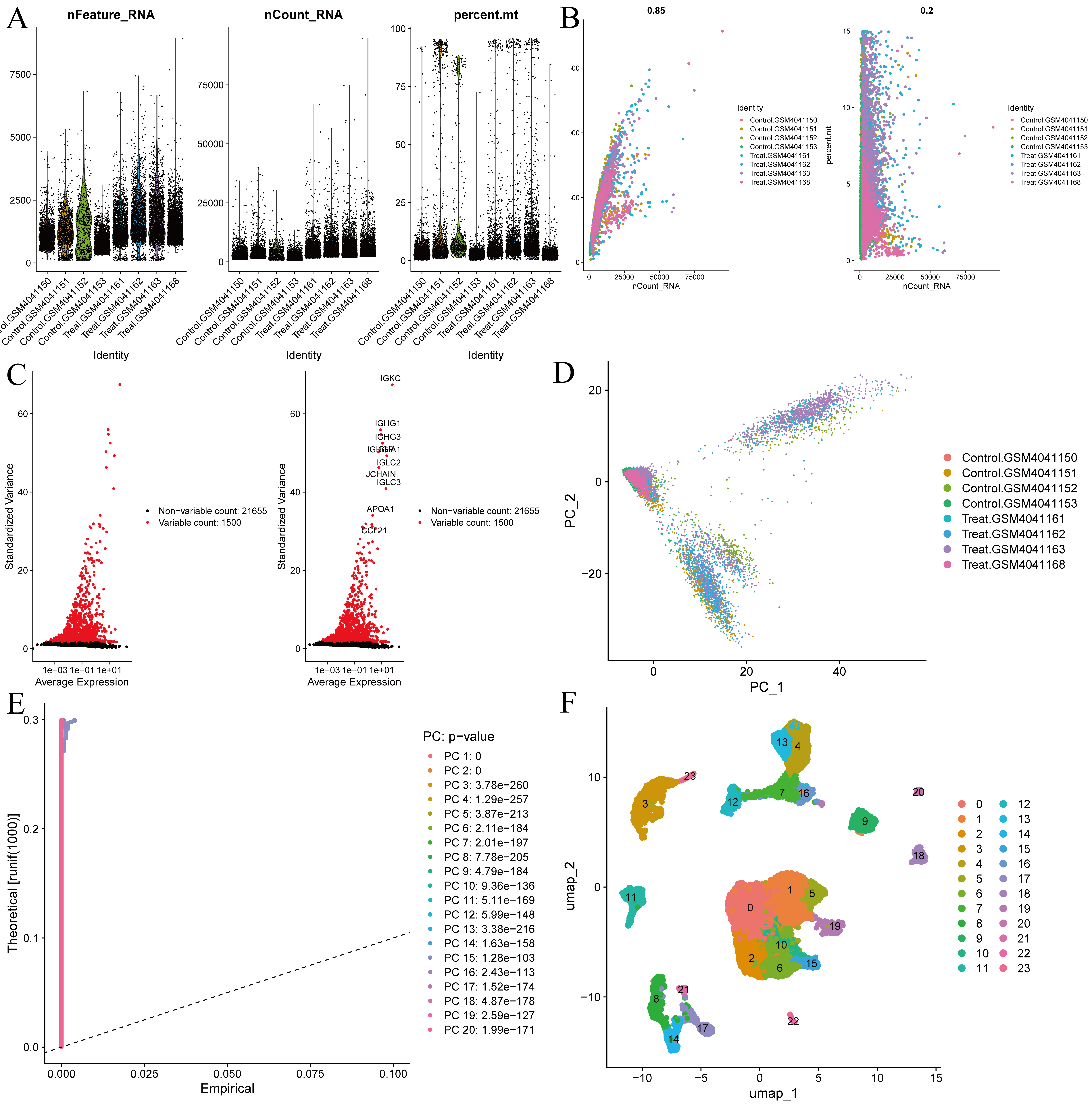


**Fig. S2**. Quality control, dimensionality reduction, clustering, and annotation of NAFLD single-cell sequencing data. (A-B) Quality control process showing the relationship between sequencing depth and relevant metrics; (C) Selection of highly variable genes for downstream analysis; (D-E) PCA dimensionality reduction analysis; (F) Identification of 24 cell clusters based on clustering analysis.


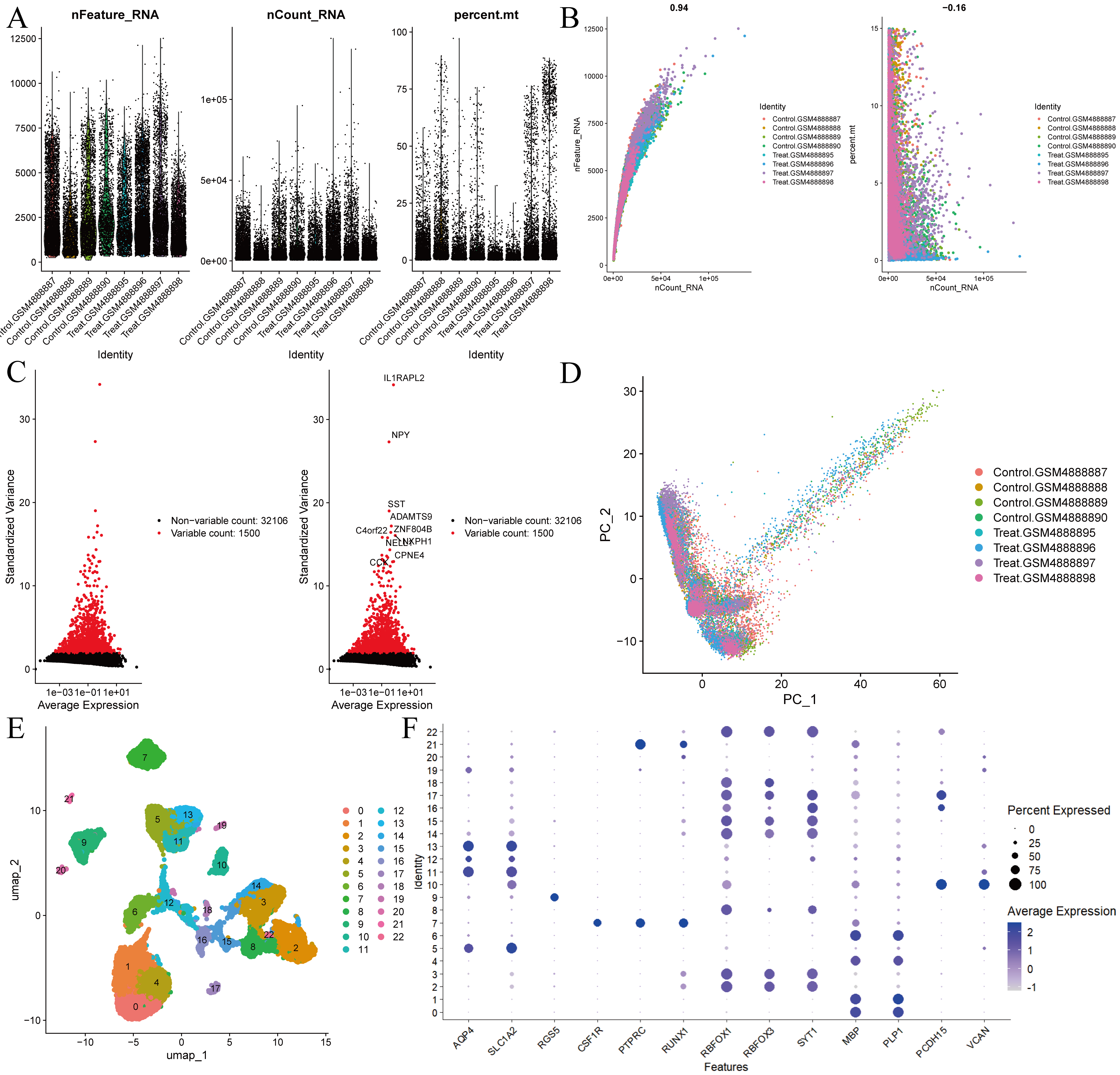


**Fig. S3.** Quality control, dimensionality reduction, clustering, and annotation of PD single-cell sequencing data. (A-B) Quality control process showing the relationship between sequencing depth and relevant metrics; (C) Selection of highly variable genes for downstream analysis; (D) PCA dimensionality reduction analysis; (E) Identification of 23 cell clusters based on clustering analysis; (F) Marker genes used for cell annotation.

**Table S1.** Predicted candidate drugs for NAFLD and PD

| **Name** | **P-value** | **Adjusted p-value** | **Odds Ratio** | **Combined score** |
| --- | --- | --- | --- | --- |
| SELICICLIB | 0.001649 | 0.004497 | 999.35 | 6403.33 |
| ETHINYL ESTRADIOL | 0.001799 | 0.004497 | 908.45 | 5741.92 |
| MESALAMINE | 0.002847 | 0.004746 | 554.97 | 3252.89 |
| GOSSYPOL | 0.007034 | 0.008792 | 216.86 | 1074.98 |
| TAMOXIFEN | 0.009122 | 0.009122 | 166.14 | 780.37 |
